# *Drosophila* Muller F Elements Maintain a Distinct Set of Genomic Properties Over 40 Million Years of Evolution

**DOI:** 10.1534/g3.114.015966

**Published:** 2015-03-04

**Authors:** Wilson Leung, Christopher D. Shaffer, Laura K. Reed, Sheryl T. Smith, William Barshop, William Dirkes, Matthew Dothager, Paul Lee, Jeannette Wong, David Xiong, Han Yuan, James E. J. Bedard, Joshua F. Machone, Seantay D. Patterson, Amber L. Price, Bryce A. Turner, Srebrenka Robic, Erin K. Luippold, Shannon R. McCartha, Tezin A. Walji, Chelsea A. Walker, Kenneth Saville, Marita K. Abrams, Andrew R. Armstrong, William Armstrong, Robert J. Bailey, Chelsea R. Barberi, Lauren R. Beck, Amanda L. Blaker, Christopher E. Blunden, Jordan P. Brand, Ethan J. Brock, Dana W. Brooks, Marie Brown, Sarah C. Butzler, Eric M. Clark, Nicole B. Clark, Ashley A. Collins, Rebecca J. Cotteleer, Peterson R. Cullimore, Seth G. Dawson, Carter T. Docking, Sasha L. Dorsett, Grace A. Dougherty, Kaitlyn A. Downey, Andrew P. Drake, Erica K. Earl, Trevor G. Floyd, Joshua D. Forsyth, Jonathan D. Foust, Spencer L. Franchi, James F. Geary, Cynthia K. Hanson, Taylor S. Harding, Cameron B. Harris, Jonathan M. Heckman, Heather L. Holderness, Nicole A. Howey, Dontae A. Jacobs, Elizabeth S. Jewell, Maria Kaisler, Elizabeth A. Karaska, James L. Kehoe, Hannah C. Koaches, Jessica Koehler, Dana Koenig, Alexander J. Kujawski, Jordan E. Kus, Jennifer A. Lammers, Rachel R. Leads, Emily C. Leatherman, Rachel N. Lippert, Gregory S. Messenger, Adam T. Morrow, Victoria Newcomb, Haley J. Plasman, Stephanie J. Potocny, Michelle K. Powers, Rachel M. Reem, Jonathan P. Rennhack, Katherine R. Reynolds, Lyndsey A. Reynolds, Dong K. Rhee, Allyson B. Rivard, Adam J. Ronk, Meghan B. Rooney, Lainey S. Rubin, Luke R. Salbert, Rasleen K. Saluja, Taylor Schauder, Allison R. Schneiter, Robert W. Schulz, Karl E. Smith, Sarah Spencer, Bryant R. Swanson, Melissa A. Tache, Ashley A. Tewilliager, Amanda K. Tilot, Eve VanEck, Matthew M. Villerot, Megan B. Vylonis, David T. Watson, Juliana A. Wurzler, Lauren M. Wysocki, Monica Yalamanchili, Matthew A. Zaborowicz, Julia A. Emerson, Carlos Ortiz, Frederic J. Deuschle, Lauren A. DiLorenzo, Katie L. Goeller, Christopher R. Macchi, Sarah E. Muller, Brittany D. Pasierb, Joseph E. Sable, Jessica M. Tucci, Marykathryn Tynon, David A. Dunbar, Levent H. Beken, Alaina C. Conturso, Benjamin L. Danner, Gabriella A. DeMichele, Justin A. Gonzales, Maureen S. Hammond, Colleen V. Kelley, Elisabeth A. Kelly, Danielle Kulich, Catherine M. Mageeney, Nikie L. McCabe, Alyssa M. Newman, Lindsay A. Spaeder, Richard A. Tumminello, Dennis Revie, Jonathon M. Benson, Michael C. Cristostomo, Paolo A. DaSilva, Katherine S. Harker, Jenifer N. Jarrell, Luis A. Jimenez, Brandon M. Katz, William R. Kennedy, Kimberly S. Kolibas, Mark T. LeBlanc, Trung T. Nguyen, Daniel S. Nicolas, Melissa D. Patao, Shane M. Patao, Bryan J. Rupley, Bridget J. Sessions, Jennifer A. Weaver, Anya L. Goodman, Erica L. Alvendia, Shana M. Baldassari, Ashley S. Brown, Ian O. Chase, Maida Chen, Scott Chiang, Avery B. Cromwell, Ashley F. Custer, Tia M. DiTommaso, Jad El-Adaimi, Nora C. Goscinski, Ryan A. Grove, Nestor Gutierrez, Raechel S. Harnoto, Heather Hedeen, Emily L. Hong, Barbara L. Hopkins, Vilma F. Huerta, Colin Khoshabian, Kristin M. LaForge, Cassidy T. Lee, Benjamin M. Lewis, Anniken M. Lydon, Brian J. Maniaci, Ryan D. Mitchell, Elaine V. Morlock, William M. Morris, Priyanka Naik, Nicole C. Olson, Jeannette M. Osterloh, Marcos A. Perez, Jonathan D. Presley, Matt J. Randazzo, Melanie K. Regan, Franca G. Rossi, Melanie A. Smith, Eugenia A. Soliterman, Ciani J. Sparks, Danny L. Tran, Tiffany Wan, Anne A. Welker, Jeremy N. Wong, Aparna Sreenivasan, Jim Youngblom, Andrew Adams, Justin Alldredge, Ashley Bryant, David Carranza, Alyssa Cifelli, Kevin Coulson, Calise Debow, Noelle Delacruz, Charlene Emerson, Cassandra Farrar, Don Foret, Edgar Garibay, John Gooch, Michelle Heslop, Sukhjit Kaur, Ambreen Khan, Van Kim, Travis Lamb, Peter Lindbeck, Gabi Lucas, Elizabeth Macias, Daniela Martiniuc, Lissett Mayorga, Joseph Medina, Nelson Membreno, Shady Messiah, Lacey Neufeld, San Francisco Nguyen, Zachary Nichols, George Odisho, Daymon Peterson, Laura Rodela, Priscilla Rodriguez, Vanessa Rodriguez, Jorge Ruiz, Will Sherrill, Valeria Silva, Jeri Sparks, Geeta Statton, Ashley Townsend, Isabel Valdez, Mary Waters, Kyle Westphal, Stacey Winkler, Joannee Zumkehr, Randall J. DeJong, Arlene J. Hoogewerf, Cheri M. Ackerman, Isaac O. Armistead, Lara Baatenburg, Matthew J. Borr, Lindsay K. Brouwer, Brandon J. Burkhart, Kelsey T. Bushhouse, Lejla Cesko, Tiffany Y. Y. Choi, Heather Cohen, Amanda M. Damsteegt, Jess M. Darusz, Cory M. Dauphin, Yelena P. Davis, Emily J. Diekema, Melissa Drewry, Michelle E. M. Eisen, Hayley M. Faber, Katherine J. Faber, Elizabeth Feenstra, Isabella T. Felzer-Kim, Brandy L. Hammond, Jesse Hendriksma, Milton R. Herrold, Julia A. Hilbrands, Emily J. Howell, Sarah A. Jelgerhuis, Timothy R. Jelsema, Benjamin K. Johnson, Kelly K. Jones, Anna Kim, Ross D. Kooienga, Erika E. Menyes, Eric A. Nollet, Brittany E. Plescher, Lindsay Rios, Jenny L. Rose, Allison J. Schepers, Geoff Scott, Joshua R. Smith, Allison M. Sterling, Jenna C. Tenney, Chris Uitvlugt, Rachel E. VanDyken, Marielle VanderVennen, Samantha Vue, Nighat P. Kokan, Kwabea Agbley, Sampson K. Boham, Daniel Broomfield, Kayla Chapman, Ali Dobbe, Ian Dobbe, William Harrington, Marwan Ibrahem, Andre Kennedy, Chad A. Koplinsky, Cassandra Kubricky, Danielle Ladzekpo, Claire Pattison, Roman E. Ramirez, Lucia Wande, Sarah Woehlke, Matthew Wawersik, Elizabeth Kiernan, Jeffrey S. Thompson, Roxanne Banker, Justina R. Bartling, Chinmoy I. Bhatiya, Anna L. Boudoures, Lena Christiansen, Daniel S. Fosselman, Kristin M. French, Ishwar S. Gill, Jessen T. Havill, Jaelyn L. Johnson, Lauren J. Keny, John M. Kerber, Bethany M. Klett, Christina N. Kufel, Francis J. May, Jonathan P. Mecoli, Callie R. Merry, Lauren R. Meyer, Emily G. Miller, Gregory J. Mullen, Katherine C. Palozola, Jacob J. Pfeil, Jessica G. Thomas, Evan M. Verbofsky, Eric P. Spana, Anant Agarwalla, Julia Chapman, Ben Chlebina, Insun Chong, I.N. Falk, John D. Fitzgibbons, Harrison Friedman, Osagie Ighile, Andrew J. Kim, Kristin A. Knouse, Faith Kung, Danny Mammo, Chun Leung Ng, Vinayak S. Nikam, Diana Norton, Philip Pham, Jessica W. Polk, Shreya Prasad, Helen Rankin, Camille D. Ratliff, Victoria Scala, Nicholas U. Schwartz, Jessica A. Shuen, Amy Xu, Thomas Q. Xu, Yi Zhang, Anne G. Rosenwald, Martin G. Burg, Stephanie J. Adams, Morgan Baker, Bobbi Botsford, Briana Brinkley, Carter Brown, Shadie Emiah, Erica Enoch, Chad Gier, Alyson Greenwell, Lindsay Hoogenboom, Jordan E. Matthews, Mitchell McDonald, Amanda Mercer, Nicholaus Monsma, Kristine Ostby, Alen Ramic, Devon Shallman, Matthew Simon, Eric Spencer, Trisha Tomkins, Pete Wendland, Anna Wylie, Michael J. Wolyniak, Gregory M. Robertson, Samuel I. Smith, Justin R. DiAngelo, Eric D. Sassu, Satish C. Bhalla, Karim A. Sharif, Tenzin Choeying, Jason S. Macias, Fareed Sanusi, Karvyn Torchon, April E. Bednarski, Consuelo J. Alvarez, Kristen C. Davis, Carrie A. Dunham, Alaina J. Grantham, Amber N. Hare, Jennifer Schottler, Zackary W. Scott, Gary A. Kuleck, Nicole S. Yu, Marian M. Kaehler, Jacob Jipp, Paul J. Overvoorde, Elizabeth Shoop, Olivia Cyrankowski, Betsy Hoover, Matt Kusner, Devry Lin, Tijana Martinov, Jonathan Misch, Garrett Salzman, Holly Schiedermayer, Michael Snavely, Stephanie Zarrasola, Susan Parrish, Atlee Baker, Alissa Beckett, Carissa Belella, Julie Bryant, Turner Conrad, Adam Fearnow, Carolina Gomez, Robert A. Herbstsomer, Sarah Hirsch, Christen Johnson, Melissa Jones, Rita Kabaso, Eric Lemmon, Carolina Marques dos Santos Vieira, Darryl McFarland, Christopher McLaughlin, Abbie Morgan, Sepo Musokotwane, William Neutzling, Jana Nietmann, Christina Paluskievicz, Jessica Penn, Emily Peoples, Caitlin Pozmanter, Emily Reed, Nichole Rigby, Lasse Schmidt, Micah Shelton, Rebecca Shuford, Tiara Tirasawasdichai, Blair Undem, Damian Urick, Kayla Vondy, Bryan Yarrington, Todd T. Eckdahl, Jeffrey L. Poet, Alica B. Allen, John E. Anderson, Jason M. Barnett, Jordan S. Baumgardner, Adam D. Brown, Jordan E. Carney, Ramiro A. Chavez, Shelbi L. Christgen, Jordan S. Christie, Andrea N. Clary, Michel A. Conn, Kristen M. Cooper, Matt J. Crowley, Samuel T. Crowley, Jennifer S. Doty, Brian A. Dow, Curtis R. Edwards, Darcie D. Elder, John P. Fanning, Bridget M. Janssen, Anthony K. Lambright, Curtiss E. Lane, Austin B. Limle, Tammy Mazur, Marly R. McCracken, Alexa M. McDonough, Amy D. Melton, Phillip J. Minnick, Adam E. Musick, William H. Newhart, Joseph W. Noynaert, Bradley J. Ogden, Michael W. Sandusky, Samantha M. Schmuecker, Anna L. Shipman, Anna L. Smith, Kristen M. Thomsen, Matthew R. Unzicker, William B. Vernon, Wesley W. Winn, Dustin S. Woyski, Xiao Zhu, Chunguang Du, Caitlin Ament, Soham Aso, Laura Simone Bisogno, Jason Caronna, Nadezhda Fefelova, Lenin Lopez, Lorraine Malkowitz, Jonathan Marra, Daniella Menillo, Ifeanyi Obiorah, Eric Nyabeta Onsarigo, Shekerah Primus, Mahdi Soos, Archana Tare, Ameer Zidan, Christopher J. Jones, Todd Aronhalt, James M. Bellush, Christa Burke, Steve DeFazio, Benjamin R. Does, Todd D. Johnson, Nicholas Keysock, Nelson H. Knudsen, James Messler, Kevin Myirski, Jade Lea Rekai, Ryan Michael Rempe, Michael S. Salgado, Erica Stagaard, Justin R. Starcher, Andrew W. Waggoner, Anastasia K. Yemelyanova, Amy T. Hark, Anne Bertolet, Cyrus E. Kuschner, Kesley Parry, Michael Quach, Lindsey Shantzer, Mary E. Shaw, Mary A. Smith, Omolara Glenn, Portia Mason, Charlotte Williams, S. Catherine Silver Key, Tyneshia C. P. Henry, Ashlee G. Johnson, Jackie X. White, Adam Haberman, Sam Asinof, Kelly Drumm, Trip Freeburg, Nadia Safa, Darrin Schultz, Yakov Shevin, Petros Svoronos, Tam Vuong, Jules Wellinghoff, Laura L. M. Hoopes, Kim M. Chau, Alyssa Ward, E. Gloria C. Regisford, LaJerald Augustine, Brionna Davis-Reyes, Vivienne Echendu, Jasmine Hales, Sharon Ibarra, Lauriaun Johnson, Steven Ovu, John M. Braverman, Thomas J. Bahr, Nicole M. Caesar, Christopher Campana, Daniel W. Cassidy, Peter A. Cognetti, Johnathan D. English, Matthew C. Fadus, Cameron N. Fick, Philip J. Freda, Bryan M. Hennessy, Kelsey Hockenberger, Jennifer K. Jones, Jessica E. King, Christopher R. Knob, Karen J. Kraftmann, Linghui Li, Lena N. Lupey, Carl J. Minniti, Thomas F. Minton, Joseph V. Moran, Krishna Mudumbi, Elizabeth C. Nordman, William J. Puetz, Lauren M. Robinson, Thomas J. Rose, Edward P. Sweeney, Ashley S. Timko, Don W. Paetkau, Heather L. Eisler, Megan E. Aldrup, Jessica M. Bodenberg, Mara G. Cole, Kelly M. Deranek, Megan DeShetler, Rose M. Dowd, Alexandra K. Eckardt, Sharon C. Ehret, Jessica Fese, Amanda D. Garrett, Anna Kammrath, Michelle L. Kappes, Morgan R. Light, Anne C. Meier, Allison O’Rouke, Mallory Perella, Kimberley Ramsey, Jennifer R. Ramthun, Mary T. Reilly, Deirdre Robinett, Nadine L. Rossi, Mary Grace Schueler, Emma Shoemaker, Kristin M. Starkey, Ashley Vetor, Abby Vrable, Vidya Chandrasekaran, Christopher Beck, Kristen R. Hatfield, Douglas A. Herrick, Christopher B. Khoury, Charlotte Lea, Christopher A. Louie, Shannon M. Lowell, Thomas J. Reynolds, Jeanine Schibler, Alexandra H. Scoma, Maxwell T. Smith-Gee, Sarah Tuberty, Christopher D. Smith, Jane E. Lopilato, Jeanette Hauke, Jennifer A. Roecklein-Canfield, Maureen Corrielus, Hannah Gilman, Stephanie Intriago, Amanda Maffa, Sabya A. Rauf, Katrina Thistle, Melissa Trieu, Jenifer Winters, Bib Yang, Charles R. Hauser, Tariq Abusheikh, Yara Ashrawi, Pedro Benitez, Lauren R. Boudreaux, Megan Bourland, Miranda Chavez, Samantha Cruz, GiNell Elliott, Jesse R. Farek, Sarah Flohr, Amanda H. Flores, Chelsey Friedrichs, Zach Fusco, Zane Goodwin, Eric Helmreich, John Kiley, John Mark Knepper, Christine Langner, Megan Martinez, Carlos Mendoza, Monal Naik, Andrea Ochoa, Nicolas Ragland, England Raimey, Sunil Rathore, Evangelina Reza, Griffin Sadovsky, Marie-Isabelle B. Seydoux, Jonathan E. Smith, Anna K. Unruh, Vicente Velasquez, Matthew W. Wolski, Yuying Gosser, Shubha Govind, Nicole Clarke-Medley, Leslie Guadron, Dawn Lau, Alvin Lu, Cheryl Mazzeo, Mariam Meghdari, Simon Ng, Brad Pamnani, Olivia Plante, Yuki Kwan Wa Shum, Roy Song, Diana E. Johnson, Mai Abdelnabi, Alexi Archambault, Norma Chamma, Shailly Gaur, Deborah Hammett, Adrese Kandahari, Guzal Khayrullina, Sonali Kumar, Samantha Lawrence, Nigel Madden, Max Mandelbaum, Heather Milnthorp, Shiv Mohini, Roshni Patel, Sarah J. Peacock, Emily Perling, Amber Quintana, Michael Rahimi, Kristen Ramirez, Rishi Singhal, Corinne Weeks, Tiffany Wong, Aubree T. Gillis, Zachary D. Moore, Christopher D. Savell, Reece Watson, Stephanie F. Mel, Arjun A. Anilkumar, Paul Bilinski, Rostislav Castillo, Michael Closser, Nathalia M. Cruz, Tiffany Dai, Giancarlo F. Garbagnati, Lanor S. Horton, Dongyeon Kim, Joyce H. Lau, James Z. Liu, Sandy D. Mach, Thu A. Phan, Yi Ren, Kenneth E. Stapleton, Jean M. Strelitz, Ray Sunjed, Joyce Stamm, Morgan C. Anderson, Bethany Grace Bonifield, Daniel Coomes, Adam Dillman, Elaine J. Durchholz, Antoinette E. Fafara-Thompson, Meleah J. Gross, Amber M. Gygi, Lesley E. Jackson, Amy Johnson, Zuzana Kocsisova, Joshua L. Manghelli, Kylie McNeil, Michael Murillo, Kierstin L. Naylor, Jessica Neely, Emmy E. Ogawa, Ashley Rich, Anna Rogers, J. Devin Spencer, Kristina M. Stemler, Allison A. Throm, Matt Van Camp, Katie Weihbrecht, T. Aaron Wiles, Mallory A. Williams, Matthew Williams, Kyle Zoll, Cheryl Bailey, Leming Zhou, Darla M. Balthaser, Azita Bashiri, Mindy E. Bower, Kayla A. Florian, Nazanin Ghavam, Elizabeth S. Greiner-Sosanko, Helmet Karim, Victor W. Mullen, Carly E. Pelchen, Paul M. Yenerall, Jiayu Zhang, Michael R. Rubin, Suzette M. Arias-Mejias, Armando G. Bermudez-Capo, Gabriela V. Bernal-Vega, Mariela Colon-Vazquez, Arelys Flores-Vazquez, Mariela Gines-Rosario, Ivan G. Llavona-Cartagena, Javier O. Martinez-Rodriguez, Lionel Ortiz-Fuentes, Eliezer O. Perez-Colomba, Joseph Perez-Otero, Elisandra Rivera, Luke J. Rodriguez-Giron, Arnaldo J. Santiago-Sanabria, Andrea M. Senquiz-Gonzalez, Frank R. Soto delValle, Dorianmarie Vargas-Franco, Karla I. Velázquez-Soto, Joan D. Zambrana-Burgos, Juan Carlos Martinez-Cruzado, Lillyann Asencio-Zayas, Kevin Babilonia-Figueroa, Francis D. Beauchamp-Pérez, Juliana Belén-Rodríguez, Luciann Bracero-Quiñones, Andrea P. Burgos-Bula, Xavier A. Collado-Méndez, Luis R. Colón-Cruz, Ana I. Correa-Muller, Jonathan L. Crooke-Rosado, José M. Cruz-García, Marianna Defendini-Ávila, Francheska M. Delgado-Peraza, Alex J. Feliciano-Cancela, Valerie M. Gónzalez-Pérez, Wilfried Guiblet, Aldo Heredia-Negrón, Jennifer Hernández-Muñiz, Lourdes N. Irizarry-González, Ángel L. Laboy-Corales, Gabriela A. Llaurador-Caraballo, Frances Marín-Maldonado, Ulises Marrero-Llerena, Héctor A. Martell-Martínez, Idaliz M. Martínez-Traverso, Kiara N. Medina-Ortega, Sonya G. Méndez-Castellanos, Krizia C. Menéndez-Serrano, Carol I. Morales-Caraballo, Saryleine Ortiz-DeChoudens, Patricia Ortiz-Ortiz, Hendrick Pagán-Torres, Diana Pérez-Afanador, Enid M. Quintana-Torres, Edwin G. Ramírez-Aponte, Carolina Riascos-Cuero, Michelle S. Rivera-Llovet, Ingrid T. Rivera-Pagán, Ramón E. Rivera-Vicéns, Fabiola Robles-Juarbe, Lorraine Rodríguez-Bonilla, Brian O. Rodríguez-Echevarría, Priscila M. Rodríguez-García, Abneris E. Rodríguez-Laboy, Susana Rodríguez-Santiago, Michael L. Rojas-Vargas, Eva N. Rubio-Marrero, Albeliz Santiago-Colón, Jorge L. Santiago-Ortiz, Carlos E. Santos-Ramos, Joseline Serrano-González, Alina M. Tamayo-Figueroa, Edna P. Tascón-Peñaranda, José L. Torres-Castillo, Nelson A. Valentín-Feliciano, Yashira M. Valentín-Feliciano, Nadyan M. Vargas-Barreto, Miguel Vélez-Vázquez, Luis R. Vilanova-Vélez, Cristina Zambrana-Echevarría, Christy MacKinnon, Hui-Min Chung, Chris Kay, Anthony Pinto, Olga R. Kopp, Joshua Burkhardt, Chris Harward, Robert Allen, Pavan Bhat, Jimmy Hsiang-Chun Chang, York Chen, Christopher Chesley, Dara Cohn, David DuPuis, Michael Fasano, Nicholas Fazzio, Katherine Gavinski, Heran Gebreyesus, Thomas Giarla, Marcus Gostelow, Rachel Greenstein, Hashini Gunasinghe, Casey Hanson, Amanda Hay, Tao Jian He, Katie Homa, Ruth Howe, Jeff Howenstein, Henry Huang, Aaditya Khatri, Young Lu Kim, Olivia Knowles, Sarah Kong, Rebecca Krock, Matt Kroll, Julia Kuhn, Matthew Kwong, Brandon Lee, Ryan Lee, Kevin Levine, Yedda Li, Bo Liu, Lucy Liu, Max Liu, Adam Lousararian, Jimmy Ma, Allyson Mallya, Charlie Manchee, Joseph Marcus, Stephen McDaniel, Michelle L. Miller, Jerome M. Molleston, Cristina Montero Diez, Patrick Ng, Natalie Ngai, Hien Nguyen, Andrew Nylander, Jason Pollack, Suchita Rastogi, Himabindu Reddy, Nathaniel Regenold, Jon Sarezky, Michael Schultz, Jien Shim, Tara Skorupa, Kenneth Smith, Sarah J. Spencer, Priya Srikanth, Gabriel Stancu, Andrew P. Stein, Marshall Strother, Lisa Sudmeier, Mengyang Sun, Varun Sundaram, Noor Tazudeen, Alan Tseng, Albert Tzeng, Rohit Venkat, Sandeep Venkataram, Leah Waldman, Tracy Wang, Hao Yang, Jack Y. Yu, Yin Zheng, Mary L. Preuss, Angelica Garcia, Matt Juergens, Robert W. Morris, Alexis A. Nagengast, Julie Azarewicz, Thomas J. Carr, Nicole Chichearo, Mike Colgan, Megan Donegan, Bob Gardner, Nik Kolba, Janice L. Krumm, Stacey Lytle, Laurell MacMillian, Mary Miller, Andrew Montgomery, Alysha Moretti, Brittney Offenbacker, Mike Polen, John Toth, John Woytanowski, Lisa Kadlec, Justin Crawford, Mary L. Spratt, Ashley L. Adams, Brianna K. Barnard, Martin N. Cheramie, Anne M. Eime, Kathryn L. Golden, Allyson P. Hawkins, Jessica E. Hill, Jessica A. Kampmeier, Cody D. Kern, Emily E. Magnuson, Ashley R. Miller, Cody M. Morrow, Julia C. Peairs, Gentry L. Pickett, Sarah A. Popelka, Alexis J. Scott, Emily J. Teepe, Katie A. TerMeer, Carmen A. Watchinski, Lucas A. Watson, Rachel E. Weber, Kate A. Woodard, Daron C. Barnard, Isaac Appiah, Michelle M. Giddens, Gerard P. McNeil, Adeola Adebayo, Kate Bagaeva, Justina Chinwong, Chrystel Dol, Eunice George, Kirk Haltaufderhyde, Joanna Haye, Manpreet Kaur, Max Semon, Dmitri Serjanov, Anika Toorie, Christopher Wilson, Nicole C. Riddle, Jeremy Buhler, Elaine R. Mardis, Sarah C. R. Elgin

**Affiliations:** a Department of Biology, Washington University in St. Louis, St. Louis, MO 63130; b Department of Biological Sciences, University of Alabama, Tuscaloosa, AL 35401; c Department of Biology, Arcadia University, Glenside, PA 19038; d Department of Biology, Adams State University, Alamosa, CO 81101; e Department of Biology, Agnes Scott College, Decatur, GA 30030; f Department of Biology, Albion College, Albion, MI 49224; g Department of Biology, Amherst College, Amherst, MA 01002; h Department of Computer Science and Mathematics, Arcadia University, Glenside, PA 19038; i Science Department, Cabrini College, Radnor, PA 19087; j Department of Biology, California Lutheran University, Thousand Oaks, CA 91360; k Department of Chemistry and Biochemistry, California Polytechnic State University, San Luis Obispo, CA 93407; l Division of Science and Environmental Policy, California State University, Monterey Bay, Seaside, CA 93950; m Department of Biology, California State University, Stanislaus, Turlock, CA 95382; n Department of Biology, Calvin College, Grand Rapids, MI 49546; o Department of Natural Sciences, Cardinal Stritch University, Milwaukee, WI 53217; p Department of Biology, College of William & Mary, Williamsburg, VA 23187; q Department of Biology, Denison University, Granville, OH 43023; r Department of Biology, Duke University, Durham, NC 27708; s Department of Biology, Georgetown University, Washington, DC 20057; t Departments of Biomedical Sciences & Cell and Molecular Biology, Grand Valley State University, Allendale, MI 49401; u Biology Department, Hampden-Sydney College, Hampden-Sydney, VA 23943; v Department of Biology, Hofstra University, Hempstead, NY 11549; w Department of Computer Science and Engineering, Johnson C. Smith University, Charlotte, NC 28216; x Department of Natural Sciences, LaGuardia Community College, Long Island City, NY 11101; y Chemistry Department, Lindenwood University, St. Charles, MO 63301; z Department of Biological and Environmental Sciences, Longwood University, Farmville, VA 23909; aa Department of Biology, Loyola Marymount University, Los Angeles, CA 90045; bb Biology Department, Luther College, Decorah, IA 52101; cc Department of Biology, Macalester College, St. Paul, MN 55105; dd Department of Mathematics, Statistics, and Computer Science, Macalester College, St. Paul, MN 55105; ee Biology Department, McDaniel College, Westminster, MD 21157; ff Department of Biology, Missouri Western State University, St. Joseph, MO 64507; gg Department of Computer Science, Math and Physics, Missouri Western State University, St. Joseph, MO 64507; hh Department of Biology & Molecular Biology, Montclair State University, Montclair, NJ 07043; ii Department of Biological Sciences, Moravian College, Bethlehem, PA 18018; jj Biology Department, Muhlenberg College, Allentown, PA 18104; kk Department of Biology, New Mexico Highlands University, Las Vegas, NM 87701; ll Department of Biology, North Carolina A&T State University, Greensboro, NC 27411; mm Biology Department, North Carolina Central University, Durham, NC 27707; nn Biology Department, Oberlin College, Oberlin, OH 44074; oo Department of Biology, Pomona College, Claremont, CA 91711; pp Department of Biology, Prairie View A&M University, Prairie View, TX 77446; qq Department of Biology, Saint Joseph’s University, Philadelphia, PA 19131; rr Department of Biology, Saint Mary’s College, Notre Dame, IN 46556; ss Department of Biology, Saint Mary’s College of California, Moraga, CA 94556; tt Department of Biology, San Francisco State University, San Francisco CA 94132; uu Biology Department, Simmons College, Boston, MA 02115; vv Department of Chemistry, Simmons College, Boston, MA 02115; ww Bioinformatics Program, St. Edward’s University, Austin, TX 78704; xx Grove School of Engineering, City College / CUNY, New York, NY10031; yy Biology Department, The City College of New York, New York, NY 10031; zz Department of Biological Sciences, The George Washington University, Washington, DC 20052; aaa Division of Biological Sciences, University of California, San Diego, La Jolla, CA 92093; bbb Department of Biology, University of Evansville, Evansville, IN 47722; ccc Department of Biochemistry, University of Nebraska–Lincoln, Lincoln, NE 68588; ddd Department of Health Information Management, University of Pittsburgh, Pittsburgh, PA 15213; eee Department of Biology, University of Puerto Rico at Cayey, Cayey, PR 00736; fff Department of Biology, University of Puerto Rico at Mayagüez, Mayagüez, PR 00680; ggg Biology Department, University of the Incarnate Word, San Antonio, TX 78209; hhh Department of Biology, University of West Florida, Pensacola, FL 32514; iii Department of Biology, Utah Valley University, Orem, UT 84058; jjj Department of Biological Sciences, Webster University, Webster Groves, MO 63119; kkk Department of Biology, Widener University, Chester, PA 19013; lll Departments of Chemistry and Biochemistry, Widener University, Chester, PA 19013; mmm Department of Biology, Wilkes University, Wilkes-Barre, PA 18766; nnn Department of Biology, William Woods University, Fulton, MO 65251; ooo Biology Department, Worcester State University, Worcester, MA 01602; ppp Department of Biology, York College / CUNY, Jamaica, NY 11451; qqq Department of Computer Science and Engineering, Washington University in St. Louis, St. Louis, MO 63130; rrr Genome Institute, Department of Genetics, Washington University School of Medicine, St. Louis, MO 63108

**Keywords:** codon bias, evolution of heterochromatin, gene size, melting characteristics, transposons

## Abstract

The Muller F element (4.2 Mb, ~80 protein-coding genes) is an unusual autosome of *Drosophila melanogaster*; it is mostly heterochromatic with a low recombination rate. To investigate how these properties impact the evolution of repeats and genes, we manually improved the sequence and annotated the genes on the *D. erecta*, *D. mojavensis*, and *D. grimshawi* F elements and euchromatic domains from the Muller D element. We find that F elements have greater transposon density (25–50%) than euchromatic reference regions (3–11%). Among the F elements, *D. grimshawi* has the lowest transposon density (particularly DINE-1: 2% *vs.* 11–27%). F element genes have larger coding spans, more coding exons, larger introns, and lower codon bias. Comparison of the Effective Number of Codons with the Codon Adaptation Index shows that, in contrast to the other species, codon bias in *D. grimshawi* F element genes can be attributed primarily to selection instead of mutational biases, suggesting that density and types of transposons affect the degree of local heterochromatin formation. F element genes have lower estimated DNA melting temperatures than D element genes, potentially facilitating transcription through heterochromatin. Most F element genes (~90%) have remained on that element, but the F element has smaller syntenic blocks than genome averages (3.4–3.6 *vs.* 8.4–8.8 genes per block), indicating greater rates of inversion despite lower rates of recombination. Overall, the F element has maintained characteristics that are distinct from other autosomes in the *Drosophila* lineage, illuminating the constraints imposed by a heterochromatic milieu.

Classically, chromatin has been demarcated into two major types based on the staining patterns in interphase nuclei. Regions that remain densely stained throughout the cell cycle are classified as heterochromatin, whereas regions that stain weakly during interphase are classified as euchromatin (Heitz 1928). Heterochromatic regions generally are late replicating and have lower rates of recombination, lower gene density, greater repeat density, greater levels of histone 3 lysine 9 di- and tri-methylation (H3K9me2/3), and associated Heterochromatin Protein 1a (HP1a) compared with euchromatic regions (reviewed in Grewal and Elgin 2007).

With an estimated size of 4.2 Mb overall, the *Drosophila melanogaster* Muller F element, (also known as the dot chromosome, or the fourth chromosome in that species) is unusual in that it appears entirely heterochromatic by most criteria, but the distal 1.3 Mb has a gene density and fraction of active genes (~50% in S2 cells) that are similar to the euchromatic regions of the *D. melanogaster* genome (Riddle *et al.* 2009, 2012). Insertion of a PEV reporter (*hsp70*-driven *white*) in most cases results in a variegating phenotype (partial silencing; see Supplemental Text in File S1), indicating that even this distal region of the F element is packaged as heterochromatin (Sun *et al.* 2004; Riddle *et al.* 2008). Subsequent high-resolution mapping of the chromatin landscape of the F element supports this conclusion (Riddle *et al.* 2012). These characteristics of the F element have made it an ideal platform for elucidating factors that are involved in heterochromatin formation and for exploring their impact on genes that are embedded in a heterochromatic domain (Elgin and Reuter 2013).

Immunofluorescent staining of polytene chromosomes with antibodies directed against H3K9me2 shows that, similar to *D. melanogaster*, the F elements of *D. erecta*, *D. mojavensis*, and *D. grimshawi* also are enriched in H3K9me2 (Figure 1, left). These enrichment patterns indicate that the F element has maintained its heterochromatic properties in species (*i.e.*, *D. mojavensis* and *D. grimshawi*) that last shared a common ancestor with *D. melanogaster* about 40 million years ago (Powell 1997; Figure 1, right).

**Figure 1:**
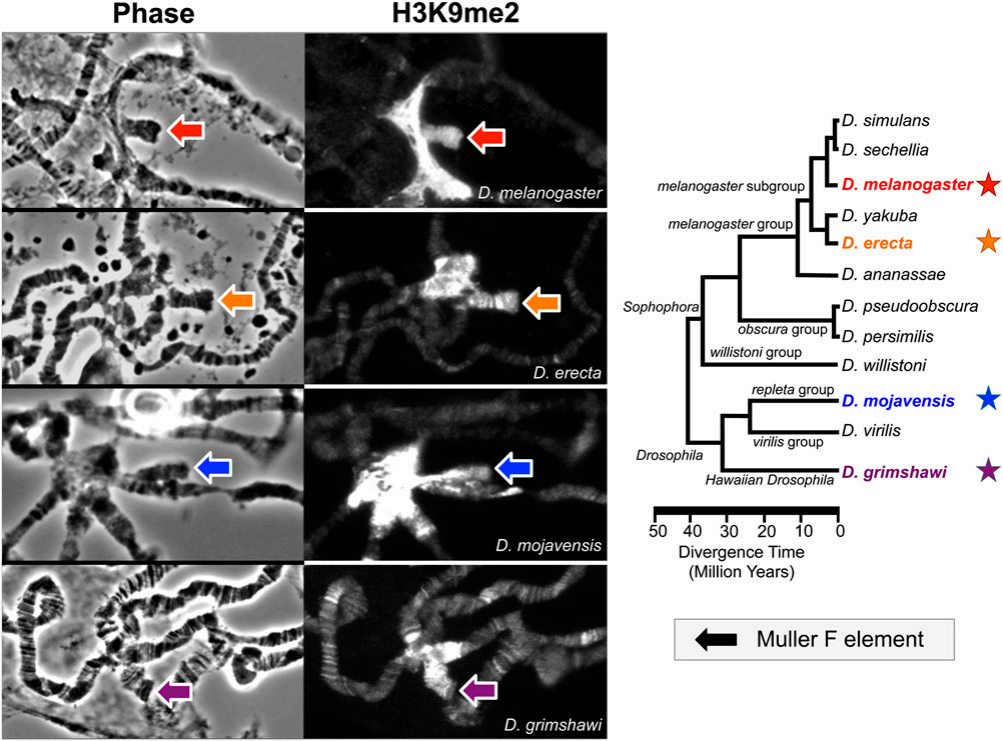
The *Drosophila* F element has maintained its heterochromatic properties in four different *Drosophila* species. (Left) Immunofluorescent staining of polytene chromosomes using H3K9me2-specific antibodies shows that the *D. melanogaster*, *D. erecta*, *D. mojavensis*, and *D. grimshawi* F elements (colored arrows) are enriched in H3K9me2 (a mark of heterochromatin). (Right) Phylogenetic tree of the *Drosophila* genomes sequenced by the *Drosophila* 12 Genomes Consortium (Powell 1997). The colored stars next to the species names in the phylogenetic tree denote the species analyzed in this study; the same color scheme is used in this and subsequent figures.

To investigate the evolution of this unusual domain, we performed comparative analyses of the repeat and gene characteristics of the F element in four *Drosophila* species. The *Drosophila* 12 Genomes Consortium (*Drosophila* 12 Genomes Consortium *et al.* 2007) and the modENCODE project (Kharchenko *et al.* 2011) have produced a large collection of genomic datasets for *D. melanogaster* and 11 other *Drosophila* species. Previous analyses of the evolution of these *Drosophila* species have relied primarily on the Comparative Analysis Freeze 1 (CAF1) draft assembly and computational (GLEAN-R) gene predictions (*Drosophila* 12 Genomes Consortium *et al.* 2007). Most of these analyses only focused on the Muller elements A–E and the properties of the F element generally have not been examined carefully.

In this study, we have built on these genomic resources by performing manual sequence improvement and gene annotation of the *D. erecta*, *D. mojavensis*, and *D. grimshawi* F elements and euchromatic reference regions derived from the Muller D elements. The D element analysis regions (referred to as “base”) are located proximal to the pericentric heterochromatin so that they have a similar topological position in the nucleus as the F element. To identify characteristics that are associated with the proximity to pericentric or telomeric heterochromatin, we also analyzed two additional euchromatic regions from the *D. erecta* D element: a 1.4-Mb region that extends further from the base of the D element (referred to as “extended”) and a 1.3-Mb region adjacent to the telomeric region of the D element (referred to as “telomeric”). [See the exact coordinates of all the analysis regions in Table S1, Genome Browser views (showing repeat density and gaps) in Figure S1, and a detailed description of how these regions were selected in File S1.]

The high-quality assemblies and gene annotations generated in this study enable us to address several questions about the evolution of the F element: What are the differences in the types and distributions of repeats among the F elements? Do F element genes exhibit different characteristics (*e.g.*, coding spans, intron sizes) compared with genes on the other autosomes? How does the low recombination rate affect codon bias, the selective pressure experienced by F element genes, and the frequency of gene movement?

Our analyses show that F element genes in both the *Sophophora* and *Drosophila* clades have maintained a set of distinct characteristics (larger gene size, lower codon bias, lower melting temperature) compared with genes on other autosomes. Most of the *D. melanogaster* F element genes (~90%) have remained on the same Muller element in all four *Drosophila* species, but there have been a large number of inversions. F elements of the species in the *Drosophila* clade (*i.e.*, *D. mojavensis* and *D. grimshawi*) exhibit different repeat distributions and gene characteristics compared to the species in the melanogaster subgroup (*i.e.*, *D. melanogaster* and *D. erecta*). F element genes generally exhibit lower codon bias and weaker positive selection compared to genes in the euchromatic reference regions; these characteristics are least pronounced in *D. grimshawi*, which also has a much lower density of the Drosophila INterspersed Element 1 (DINE-1) transposon. Despite these differences, our analyses show that F element genes in all four species generally share a common set of characteristics that presumably reflect the local environment and could contribute to their ability to function in a heterochromatic domain.

## Materials and Methods

### General overview

Sequence improvement and gene annotation of the three *Drosophila* species studied here were organized using the framework provided by Genomics Education Partnership (Shaffer *et al.* 2010). Additional details for some of the analysis protocols are available in File S1. We have set up an instance of the University of California, Santa Cruz (UCSC) Genome Browser (Kent *et al.* 2002) to facilitate the visualization and access to the improved sequences and gene annotations produced in this study (available at http://gander.wustl.edu). The improved sequences and annotations are also available in File S9.

Most of the data conversions were performed with the use of tools in the Kent source utilities (part of the UCSC Genome Browser source tree; Kent *et al.* 2002). BEDTools was used to identify intersections and unions among genomic features and to manipulate BED files (Quinlan and Hall 2010). Custom scripts were used to facilitate data conversion and analysis. The analyses were run on a Dell Precision T5400 Linux server (with 8 Xeon processors and 8GB of RAM) and a MacBook Pro laptop (with an Intel Core i7 processor and 8GB of RAM). Some of the analyses were run in parallel using GNU Parallel (Tange 2011).

### Immunofluorescent staining of polytene chromosomes

The *D. erecta* (14021−0224.01), *D. mojavensis* (15081−1352.22), and *D. grimshawi* (15287−2541.00) stocks were obtained from the *Drosophila* Species Stock Center at the University of California, San Diego. The protocol for the immunofluorescent staining of polytene chromosomes from *Drosophila* third instar larval salivary glands has been described previously (Stephens *et al.* 2004). An anti-H3K9me2 rabbit polyclonal antibody (Upstate 07-441) was used at a dilution of 1:250. Secondary antibody labeled with Alexa-Fluor 594 (red) was used at a 1:750 dilution (Invitrogen, catalog number A-11012). Formaldehyde fixation times were 12 min, with the exception of *D. grimshawi* salivary glands, which were fixed for 10 min before squashing and staining.

### Sequence improvement

The *D. mojavensis* and *D. grimshawi* CAF1 assemblies produced by the *Drosophila* 12 Genomes Consortium were retrieved from the AAA: 12 *Drosophila* Genomes web site (http://rana.lbl.gov/drosophila/). The placements of the fosmid end reads were specified in the *reads.placed* file in each CAF1 assembly. The F and D element scaffolds were partitioned into a list of overlapping fosmids based on the *reads.placed* file for each species. This set of fosmids was obtained from the *Drosophila* Genomics Resource Center at Indiana University and used as templates for sequencing reactions. However, because many of the fosmid clones used to construct the original *D. grimshawi* CAF1 assemblies were unavailable from the *Drosophila* Genomics Resource Center, we could only improve approximately 90% of the *D. grimshawi* F element. Hence the analysis of this region was performed on a mosaic of the original CAF1 assembly and improved regions.

The overall sequence improvement protocol has previously been described (Slawson *et al.* 2006; Leung *et al.* 2010). Reads placed in each fosmid region were retrieved from the National Center for Biotechnology Information Trace Archive (http://www.ncbi.nlm.nih.gov/Traces/home/) and assembled using the Phred, Phrap, and Consed software package (Ewing and Green 1998; Gordon *et al.* 1998). In collaboration with the Genome Institute at Washington University, we improved each fosmid project by identifying and resolving misassemblies as well as designing additional sequencing reactions to resolve gaps and low quality regions. These fosmid projects were improved to a sequence improvement standard similar to the one used by the mouse genome project (Mouse Genome Sequencing Consortium *et al.* 2002). To ensure the correctness of the final assembly, inconsistent mate pairs within each fosmid project were resolved and restriction digests were used to confirm the final assembly. Each fosmid was digested with four restriction enzymes (*i.e.*, *Eco*RI, *Eco*RV, *Hin*dIII, and *Sac*I). The fragment sizes of the *in silico* digests of the final consensus sequence must be in congruence with the fragment sizes of at least two of the actual restriction digests to meet the standard. Each fosmid project was completed by at least two students independently; experienced undergraduates worked with the Genomics Education Partnership (GEP) staff to reconcile the results and produce the final consensus sequence.

To identify differences between the CAF1 and improved sequences, the CAF1 sequence was soft-masked using WindowMasker with default parameters. The improved sequences were compared against the original CAF1 sequence using MegaBLAST (Morgulis *et al.* 2008) with an E-value threshold of 1e-5. The UCSC Chain and Net protocol (Kent *et al.* 2003) was then applied to the MegaBLAST alignments. The Net alignments were converted into PSL and BED formats to facilitate analysis of the differences between the two assemblies.

### Repeat analysis

WindowMasker (Morgulis *et al.* 2006) was run on the different analysis regions using default parameters and the results were converted into BED format using custom Perl scripts. Tallymer (Kurtz *et al.* 2008) was used to estimate k-mer frequencies in the different analysis regions. Each genome assembly was indexed using *mkindex* and the *occratio* program was used to determine the distributions of unique k-mers. The count of each 13-mer was generated using the *search* program in Tallymer. Tandem repeats were identified using Tandem Repeats Finder (Benson 1999) with the following parameters: Match = 2, Mismatch = 7, Delta = 7, Match Probability = 80, Mismatch Probability = 10, Minscore = 50, and MaxPeriod = 2000. Simple repeats and low complexity regions were identified using tantan (Frith 2011) with default parameters (-r = 0.005), and the results were reported in BED format (-f 3). The distribution of dinucleotide repeats was determined using a Perl script that iterates from a dinucleotide repeat size of 2−100. Each dinucleotide repeat was searched against the analysis regions and the (potentially overlapping) matches were tabulated and plotted using Microsoft Excel.

### Transposon analysis

The protocols used to construct and classify the species-specific transposon libraries are described in File S1. The *Drosophila* RepBase repeat library (release 17.07) was obtained from RepBase (Jurka *et al.* 2005). The ReAS repeat library (version 2) was obtained from the FlyBase FTP site at ftp://ftp.flybase.net/genomes/aaa/transposable_elements/ReAS/v2/consensus_fasta/.

RepeatMasker (Smit *et al.* 1996) (version open-3.4.0) was run on the analysis regions using the *cross_match* search engine at the most sensitive (-s) setting, without masking low complexity or simple repeats (-nolow). Transposon fragments identified by RepeatMasker were converted into BED format using custom scripts for subsequent analysis. Overlapping transposon fragments identified by RepeatMasker were merged together using BEDTools only if the overlapping repeats had the same repeat class. Repeat density was calculated using a sliding window of 1 kb with a step size of 500 bp.

### Gene annotations

This comparative analysis used the high-quality *D. melanogaster* gene annotations (release 5.50) produced by FlyBase as reference (Marygold *et al.* 2013). The annotation protocol has been described previously (Shaffer *et al.* 2010). GEP students annotated each fosmid by using computational evidence organized on an instance of the UCSC Genome Browser (Kent *et al.* 2002) set up by the GEP staff. The computational evidence included sequence similarity to *D. melanogaster* proteins as well as predictions from multiple *ab initio* and evidence-based gene predictors. For species with RNA-Seq data, additional evidence tracks such as RNA-Seq read coverage, splice junction predictions from TopHat (Trapnell *et al.* 2009) and assembled transcripts from Cufflinks (Trapnell *et al.* 2010) were also made available. See File S1 for additional details on the protocol used to construct the RNA-Seq transcriptome and predicted protein libraries for each species.

The GEP has developed a set of annotation guidelines (Annotation Instruction Sheet) to standardize the treatment of annotations that are ambiguous because of insufficient evidence. These annotation guidelines and additional resources supporting the GEP annotation protocol are available on the GEP web site (http://gep.wustl.edu).

Each annotation project was completed independently by at least two GEP students. The GEP staff supervised students who reconciled the submitted annotations using the Apollo Genome Annotation Curation Tool (Lewis *et al.* 2002). These reconciled gene annotations were mapped back to the improved genomic scaffolds and were incorporated into the GEP UCSC Genome Browser (available through the “GEP Genes” track, http://gander.wustl.edu). The GEP staff reviewed these gene models in the context of all the available evidence tracks to resolve any remaining annotation issues.

The *D. erecta*, *D. mojavensis*, and *D. grimshawi* GLEAN-R gene annotations (Release 1.3) produced by the *Drosophila* 12 Genomes Consortium were compared to the annotations produced here. The GLEAN-R annotations were obtained from FlyBase (available at http://flybase.org/static_pages/downloads/bulkdata7.html) and converted into BED format using custom scripts. We used BLAT (Kent 2002) with default parameters to map the *D. mojavensis* and *D. grimshawi* GLEAN-R gene predictions against the improved assemblies because the underlying genomic sequences for these two species have changed due to the sequence improvements reported here. Utilities in BEDTools (Quinlan and Hall 2010) and custom scripts were then used to compare the GLEAN-R predictions with our gene annotations.

### Analysis of gene characteristics

The GEP gene annotations are in BED format, and most of the gene characteristics (*e.g.*, gene size, coding exon size) were determined using BEDTools (Quinlan and Hall 2010) and custom scripts. When calculating the coding exon sizes for the first and last coding exons, only the translated portion of the exon was included even though the transcribed exon may be larger because of untranslated regions. The gene characteristics of the most comprehensive isoform for each gene were imported into R (version 3.0.2) for subsequent analysis and visualization of the results.

Violin plots of the different gene characteristics were generated by the *vioplot* function in the R vioplot package. The Kruskal-Wallis Rank Sum Test was performed using the *kruskal.test* function in R (R Core Team 2013). The *kruskalmc* function in the pgirmess package was used to perform the multiple comparison tests after Kruskal-Wallis.

### Codon bias analysis

The Effective Number of Codons (Nc) and the Codon Adaptation Index (CAI) for each gene in the analysis regions were determined using the chips and the cai programs in the EMBOSS package (Rice *et al.* 2000), respectively. Typically, highly expressed genes are used as the reference set when calculating CAI because they are under the strongest translational selection and would typically show a strong preference for a subset of transfer RNAs (Rocha 2004). Because expression data were unavailable for some of the species used in this study, we used the program scnRCA (O’Neill *et al.* 2013) to analyze all of the GLEAN-R predictions to construct the species-specific reference gene set that exhibits the dominant codon bias for each species. The scnRCA parameters used to construct the reference gene sets were as follows: -i r -g true -d 2.0 -p 1.0 -m -1.

The codon frequency table for each species was created by analyzing the species-specific reference gene set with the cusp program in the EMBOSS package. The species-specific codon usage tables were then used in the cai program (via the -cfile parameter) to calculate the CAI value for each gene. The violin plots and Kruskal-Wallis Tests were created using the same procedure as described in the “Analysis of gene characteristics” section.

Heat maps of codon bias for each gene in the analysis regions were created using the *heatmap.2* function in the R package gplots. The dendrograms next to the heat maps were created using Ward hierarchical clustering with Euclidean distance.

### Nc *vs.* CAI scatterplots

The codon bias statistics for each gene were calculated as described above and the results were imported into R to produce the Nc *vs.* CAI scatterplots. We then applied locally estimated scatterplot smoothing (LOESS) to identify the major trends in the scatterplots (Cleveland and Devlin 1988). The span parameter for the LOESS regression line was determined by generalized cross-validation (criterion = gcv, family = symmetric) using the *loess.as* function in the R package fANCOVA.

#### Melting temperature metagene profile:

Because the transcription start sites have not been identified in *D. erecta*, *D. mojavensis*, and *D. grimshawi* gene annotations, we used the coding span (*i.e.*, from start codon to stop codon, including introns) and the 2 kb upstream and downstream of the coding spans as a first approximation for this analysis. The melting temperatures were determined by the dan tool in the EMBOSS package using a sliding window of 9 bp (windowsize = 9) and a step size of 1 (shiftincrement = 1) with the following parameters: dnaconc = 50, saltconc = 50, mintemp = 55. The results were converted into BigWig format (Kent *et al.* 2010) for subsequent analysis.

Melting temperatures for the coding spans were normalized to 3 kb using bigWigSummary (part of the Kent source utilities). Melting temperatures for the normalized 3 kb region and the 2 kb flanking regions were imported into R and the standard graphics *plot* function in R was used to produce the metagene profiles.

### Distance–Distance plots of gene characteristics

To determine whether any subset of F element genes has characteristics that differ from those of the group of genes as a whole, we constructed Distance–Distance plots for each F element separately using the rrcov package in R. Eight characteristics of the most comprehensive isoform of each gene were used in this analysis: coding span (bp from start to stop codon, including introns); intron repeat size (total size of all transposon fragments within introns); size of coding regions (sum of all coding exons in bp); number of coding exons; median size (in bp) of coding exons; median size (in bp) of introns; and Nc and CAI (calculated as described previously).

Using these eight gene characteristics, we calculated the classical Mahalanobis distance (MD) for each gene. MD measures the difference between the characteristics of each gene and the centroid (which is derived from the multivariate distribution of the characteristics of all F element genes). Unlike Euclidean distances, MD accounts for the variance of each gene characteristic and the covariance among the eight gene characteristics. The magnitude of MD corresponds to the dissimilarity of the characteristics of each gene compared to the centroid (*i.e.*, large MD indicates that the gene has very different characteristics compared to the rest of the genes in the dataset).

However, because MD is sensitive to extreme outliers, we also calculated the robust Mahalanobis distance (RD) using the Stahel-Donoho estimator (sde). This robust estimator mitigates the impact of outliers on MD by assigning a weight to each gene based on its outlyingness (calculated using projection pursuit; (Van Aelst *et al.* 2012). Hence a scatterplot of MD *vs.* RD (*i.e.*, Distance–Distance plot) can be used to identify additional outliers that were masked by classical MD.

To create the Distance–Distance plots, the gene characteristics were normalized using the *scale* function in R because the different variables have values that differ by orders of magnitude (*e.g.*, gene span *vs.* CAI). The *CovRobust* function in the rrcov package was used to calculate the robust distances (with the parameter “sde”). Plots of the RD *vs.* the MD were produced using the generic *plot* command in R (with the parameter “which=‘dd’”). Points were considered to be outliers if their values were greater than the square root of the 97.5% quantile of the χ^2^ distribution with 8 degrees of freedom (*i.e.*, 4.19).

### Whole-genome alignments

To facilitate analysis of the wanderer genes (genes present on the F element in one species and on another Muller element in a different species), we produced a set of whole-genome alignments for *D. melanogaster*, *D. yakuba*, *D. erecta*, *D. mojavensis*, *D. virilis*, and *D. grimshawi*. (The Chain and Net alignments are available on the GEP UCSC Genome Browser, http://gander.wustl.edu.) Repeats in each genome were soft masked and the genome assemblies were aligned against each other using LAST (Kiełbasa *et al.* 2011) with default parameters followed by the UCSC Chaining and Netting protocol (Kent *et al.* 2003).

## Results

### Improved F and D element assemblies and gene annotations

#### Sequence improvement:

Previous studies have shown that the *Drosophila* F elements have a greater repeat density than the other autosomes (Leung *et al.* 2010), which could lead to a greater frequency of gaps and misassemblies. These assembly issues could introduce substantial bias into the analysis of genome characteristics (Salzberg and Yorke 2005). Quality assessments (see File S1) of the CAF1 assemblies (*Drosophila* 12 Genomes Consortium *et al.* 2007) led us to improve the *D. mojavensis* F element, the *D. grimshawi* F element, and the *D. mojavensis* euchromatic reference region from the D element to a quality standard that is similar to those used for the mouse genome project. As part of this sequence improvement standard, we resolved inconsistent mate pairs within each assembly and confirmed each assembly using restriction digests (see the section *Materials and Methods* for details). These experimental data provided additional confirmation of the accuracy of the final F element assemblies, and enabled us to perform genomic analysis of the F elements with high confidence, ensuring accuracy (in particular) in the repeat and gene movement analyses.

Collectively, sequence improvement of the *D. mojavensis* and *D. grimshawi* analysis regions covered a total of approximately 3.8 Mb (1.7 Mb from the *D. mojavensis* F element, 1.1 Mb from the *D. grimshawi* F element, and 1.0 Mb from the *D. mojavensis* D element), closing 72 of 86 gaps and adding a total of 44,468 bases (Table S2A). Alignments between the CAF1 and the improved regions identified a total of 309 changes; 127 (41.1%) of these changes are single base substitutions, insertions, or deletions, while the remaining changes are more substantial (Table S2B). Detailed alignments between the CAF1 and the improved regions are available through the “*D. mojavensis* CAF1 Difference” and “*D. grimshawi* CAF1 Difference” tracks on the GEP UCSC Genome Browser (http://gander.wustl.edu).

**Figure 2:**
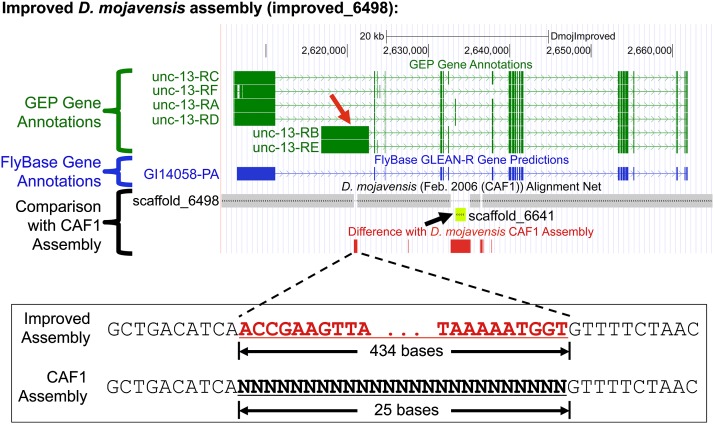
Sequence improvement of the *D. mojavensis* F element scaffold. One of the gaps in the *D. mojavensis* CAF1 assembly is located within the initial coding exon of the B and E isoforms of the putative ortholog of *unc-13* in *D. mojavensis* (red arrow). The improved assembly added 434 bases to resolve the 25-bp gap in this region (bottom) and allows us to produce annotation for the entire coding exon. Another gap was resolved by incorporating a 1.2-kb scaffold (scaffold_6641, chartreuse yellow rectangle) from the CAF1 assembly into the improved F element assembly (black arrow). This scaffold contains an internal coding exon for the A and D isoforms of *unc-13*. The remaining gaps and low quality regions were resolved by additional sequencing. Changes between the CAF1 and the improved assemblies are summarized in the “Difference with *D. mojavensis* CAF1 Assembly” track (red rectangles). The “GEP Gene Annotations” track (green) shows the manual gene annotations for all the isoforms of *unc-13* in *D. mojavensis* based on the improved sequence. The “FlyBase Gene Annotations” evidence track (blue) shows the GLEAN-R gene predictions currently maintained by FlyBase.

An example of the improvement achieved is shown for the region surrounding the GLEAN-R annotation GI14058-PA (a putative ortholog of the *D. melanogaster unc-13* gene) in *D. mojavensis*; this illustrates how the improved assemblies enabled us to produce more accurate gene models for the *D. mojavensis* F element (Figure 2).

#### Manual gene annotations:

We also constructed manually curated gene models, including all isoforms, for each of the analysis regions. Because of the large evolutionary distance among *D. melanogaster*, *D. mojavensis*, and *D. grimshawi* and the limited expression data available, this analysis only focuses on the coding regions of genes. (See the section *Materials and Methods* and File S1 for detailed description of the annotation protocol.) The manual annotation process also allows us to identify potential annotation errors in *D. melanogaster* (*e.g.*, *rdgC* as described in File S1).

Collectively, we annotated a total of 878 genes (1619 isoforms). A summary of the changes in the number of isoforms and coding exons, as well as descriptions of other noncanonical features (*e.g.*, novel GC donor sites) compared with *D. melanogaster* (release 5.50) is available in File S2. Overall, 58% (552/947) of the GLEAN-R gene predictions match our annotation of the most comprehensive isoform (*i.e.*, the isoform with the largest coding region, Table S3A), and 85% (3648/4287) of the coding exons predicted by GLEAN-R match the coding exons in the most comprehensive isoform (Table S3B).

Although a similar percentage of the coding exons predicted by GLEAN-R match our annotations in both the F and D elements (80.7–82.8%), a substantially lower percentage of the GLEAN-R gene models match our annotations on the *D. mojavensis* and *D. grimshawi* F elements (32.1% and 39.1%, respectively) than on the D elements (57.6% and 58.0%, respectively). Many of the differences between the GLEAN-R predictions and our annotations on the *D. mojavensis* and *D. grimshawi* F elements can be traced to improvement of the underlying sequence (*e.g.*, *unc-13* in Figure 2). Hence, the lower percentage of GLEAN-R gene models that match our annotations can primarily be attributed to the higher rate of assembly problems in the CAF1 assemblies for the *D. mojavensis* and *D. grimshawi* F elements. Our results show that manual sequence improvement and gene annotation can improve over half of the gene models in regions with high repeat density.

### F elements consistently show high repeat density but vary in repeat composition

**Figure 3:**
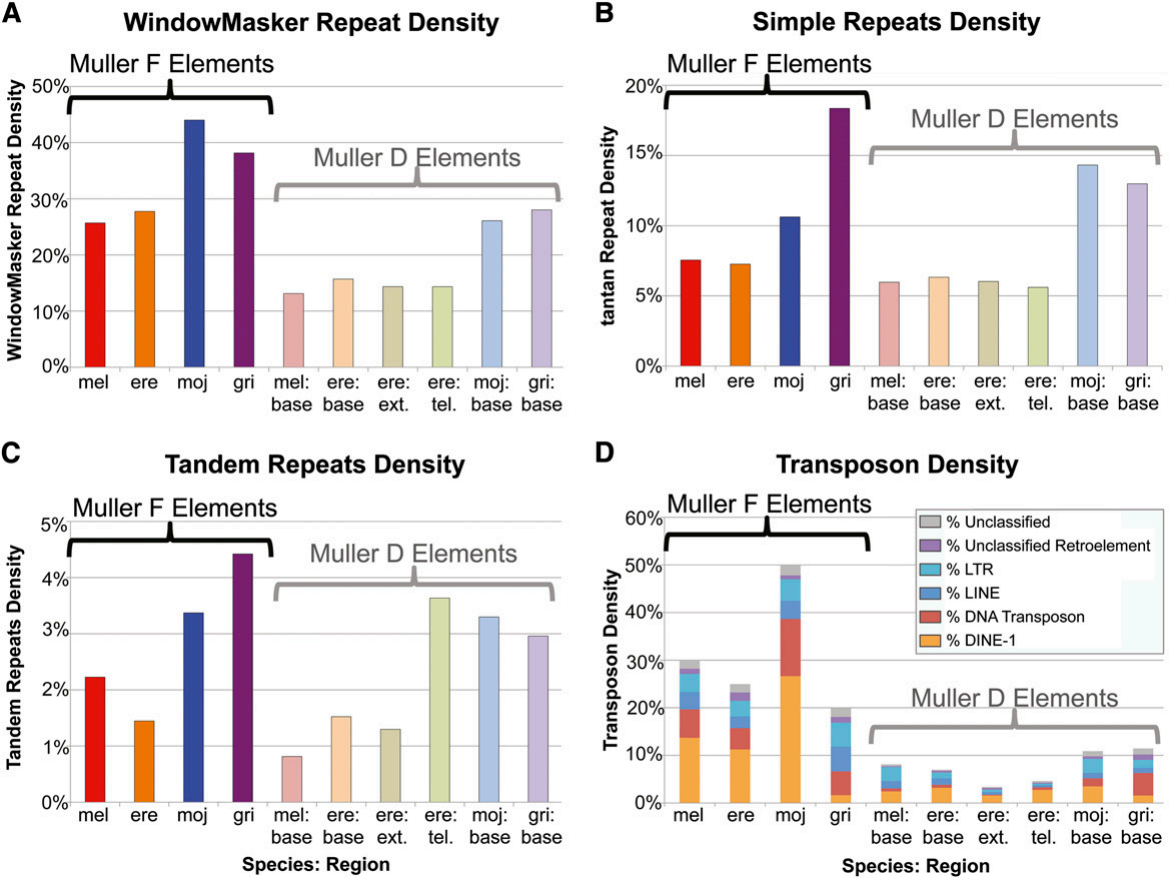
The repetitive element landscapes of the F and the base of the D elements in *D. melanogaster* (red), *D. erecta* (orange), *D. mojavensis* (blue), and *D. grimshawi* (purple). (A) WindowMasker analysis (low complexity repeats and transposons); (B) tantan analysis (simple and low complexity repeats); (C) Tandem Repeats Finder; (D) RepeatMasker analysis (transposon density). Within each species, the F element generally shows a higher repeat density (particularly transposable elements) than the euchromatic reference regions from the D elements. Except for tandem repeats, the base (light orange), extended (olive), and telomeric (green) regions from the *D. erecta* D element generally show similar repeat density.

The most striking difference between the *D. melanogaster* F element and the other autosomes is its high density of repeats, primarily remnants of transposable elements (Bergman *et al.* 2006; Riddle *et al.* 2009). To obtain an overview of the repetitive element landscape of F elements in the four *Drosophila* species, we analyzed the types and distribution of repeats using four different approaches: WindowMasker, tantan, Tandem Repeats Finder, and RepeatMasker with species-specific transposon libraries (Figure 3). (Detailed repeat statistics are available in File S3 and File S4.)

#### WindowMasker analysis shows the F elements have high repeat density:

To obtain an overview of the total repeat content, we tabulated the total number of bases masked by WindowMasker for each of the analysis regions. Unlike other repeat finding tools, WindowMasker relies only on the genomic sequence to identify over-represented sequences that correspond to low complexity sequences, simple repeats, or transposable elements, which makes it an ideal tool for analyzing the repeat contents of genomes without comprehensive repeat libraries (Morgulis *et al.* 2006). The results show that F elements consistently exhibit higher repeat densities than their corresponding euchromatic reference regions (D elements) in all four species (Figure 3A). *D. mojavensis* and *D. grimshawi* have higher repeat densities than *D. melanogaster* and *D. erecta* in both the F elements and the D elements. In fact, the *D. mojavensis* and *D. grimshawi* D elements have repeat densities that are similar to those of the *D. melanogaster* and *D. erecta* F elements.

**Figure 4:**
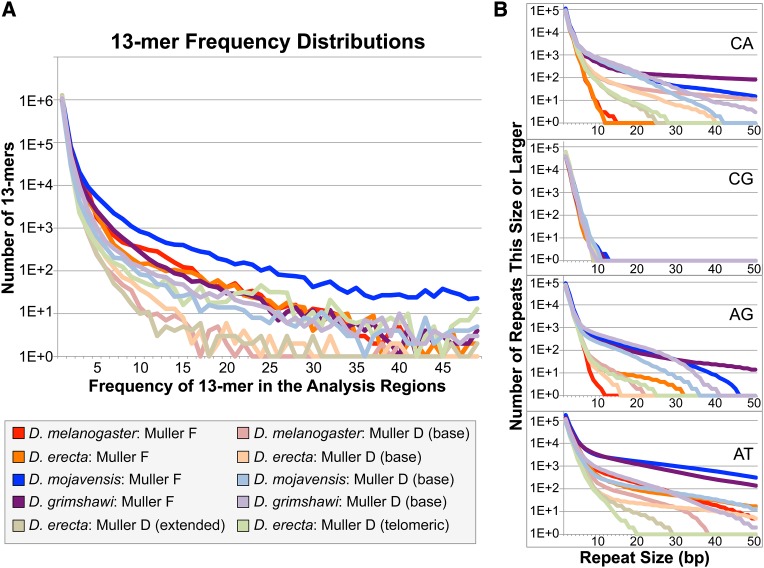
Distributions of 13-mers and dinucleotide repeats in the regions analyzed. (A) Consistent with the WindowMasker results, more 13-mers are found to be repeated (present at a higher frequency) on the *D. mojavensis* F element (dark blue line) than the other analysis regions. The genomic sequence in each analysis region is partitioned into overlapping 13-mers and the frequency of each 13-mer is tabulated using Tallymer. The values on the x-axis correspond to the number of times that a particular 13-mer is found in the analysis region whereas the y-axis correspond to the total number of 13-mers (of all sequences) that appear at each frequency. For example, approximately 10^6^ 13-mers appear only once in each analysis region. (B) Cumulative dinucleotide repeats analysis shows a higher frequency of dinucleotide repeats on the *D. mojavensis* and *D. grimshawi* F elements (dark blue and purple lines, respectively) than on the *D. melanogaster* and *D. erecta* F elements (dark red and orange lines, respectively). A pseudocount of one has been added to the cumulative distribution plots in order to show a continuous distribution in the semi-log plot.

To better understand the composition of the repeats identified by WindowMasker, we used Tallymer (Kurtz *et al.* 2008) to analyze the frequency of short sequences (*i.e.*, words) in each analysis region. A more repetitive region requires a larger word size in order to achieve the same percentage of words that are unique compared with a less repetitive region (Chor *et al.* 2009). Tallymer analysis shows that approximately 95% of the 13-mers (*i.e.*, sequences with a length of 13) are unique in the euchromatic reference regions (Table S4). In congruence with the WindowMasker results, which show that the *D. mojavensis* F element has the highest repeat density, we find that more 13-mers appear at a greater frequency on the *D. mojavensis* F element than in the other analysis regions. In contrast, most of the 13-mers at the base of the *D. melanogaster* and *D. erecta* D elements occur at low frequencies. The Tallymer analysis also shows that the *D. grimshawi* F and D elements have the most similar distributions of 13-mers (*i.e.*, the most similar repeat density) among the four species (Figure 4A).

Examination of the 13-mers identified by Tallymer shows that many of the 13-mers that appear at a high frequency in *D. mojavensis* and *D. grimshawi* contain AT and CA dinucleotide repeats. Analyses of the distribution of dinucleotide repeats show that CA dinucleotide repeats are shorter on the *D. melanogaster* and *D. erecta* F elements, but longer on the *D. mojavensis* and *D. grimshawi* F elements, than in the euchromatic reference regions (Figure 4B). Thus, while low density of CA repeats was previously associated with the F element in *D. melanogaster* (Pardue *et al.* 1987), this does not seem to hold in general. The *D. mojavensis* and *D. grimshawi* F elements are also enriched in AT dinucleotide repeats compared with those of *D. melanogaster* and *D. erecta*. The lack of CG repeats in both the F and D elements is also striking (see the *Discussion* section).

#### Simple and low complexity repeats are particularly abundant on the D. grimshawi F element:

The tantan analysis (Frith 2011) shows that *D. mojavensis* and *D. grimshawi* have a greater density of simple and low complexity sequences in both the F element and the euchromatic reference regions compared with the corresponding regions in *D. melanogaster* and *D. erecta* (Figure 3B). The analysis also reveals some species-specific differences: simple and low complexity repeats appear to contribute the most to the repeat density of the *D. grimshawi* genome. The *D. grimshawi* F element has a substantially greater density of simple and low complexity repeats (18%) compared with the F elements of the other species examined (7–11%). In contrast to the other species, the *D. mojavensis* F element shows a lower density of simple and low complexity repeats compared to its euchromatic reference region (11% *vs.* 14%).

#### Tandem repeats show a skewed distribution on the D. erecta D element:

Tandem repeats may play a particular role in genome rearrangement and regulation of gene expression (Sinha and Siggia 2005; Farré *et al.* 2011). For this analysis, tandem repeats are defined as regions with a minimum size of 25 bases and a maximum period of 2000 (see the section *Materials and Methods* for the complete list of search parameters). Results from Tandem Repeats Finder (Benson 1999) show that the *D. mojavensis* and *D. grimshawi* F elements and their euchromatic reference regions have a higher density of tandem repeats than the corresponding regions in *D. melanogaster* and *D. erecta* (Figure 3C). Although the base and the extended regions of the *D. erecta* D element both show a low density of tandem repeats, the analysis region near the telomere shows a high density, as do the euchromatic reference regions in *D. mojavensis* and *D. grimshawi*. A skew to a greater density of tandem repeats toward the telomere is apparent in a sliding window analysis of the *D. erecta* D element as a whole. In contrast, the *D. melanogaster* D element does not show the same skew in the density of tandem repeats (Figure S2).

#### Recent expansion of DINE-1 transposons leads to high transposon density on the D. mojavensis F element:

Transposons may play an important role in targeting heterochromatin formation (Grewal and Elgin 2007). Because many transposons are species-specific, we constructed transposon libraries for each species and then used RepeatMasker (Smit *et al.* 1996) to identify transposon remnants in each analysis region. (See File S1 for the protocols used to construct and classify the species-specific transposon libraries, and File S4 for transposon density estimates using different species-specific transposon libraries.) Among the F elements, *D. mojavensis* has the highest transposon density (~50%) whereas *D. grimshawi* has the lowest (~20%). Strikingly, ~53% of the transposon fragments on the *D. mojavensis* F element show sequence similarity to DINE-1 elements.

The RepeatMasker results are generally in concordance with the WindowMasker results (Figure 3D): F elements have a greater transposon density compared with the euchromatic reference regions (D elements). In some cases the transposon density estimate is higher than the total repeat density estimate by WindowMasker (*e.g.*, *D. mojavensis* F element). This discrepancy is primarily caused by the difficulty associated with precisely defining the boundaries of each repeat copy (Bao and Eddy 2002).

Although the WindowMasker analysis (Figure 3A) shows that the *D. grimshawi* and *D. mojavensis* F elements have a similar repeat density (38% and 44%, respectively), the RepeatMasker analysis (Figure 3D) shows that the *D. grimshawi* F element has a much lower density of transposons than the *D. mojavensis* F element (20% and 50%, respectively). This difference can primarily be attributed to the density of DINE-1 elements (2% in *D. grimshawi vs.* 27% in *D. mojavensis*) and DNA transposons (5% *vs.* 12%). In particular, DINE-1 (a helitron) accounts for 53% of the *D. mojavensis* F element transposon fragments but only 8% of the transposon fragments on the *D. grimshawi* F element (Figure S3). DINE-1 elements account for approximately half of all transposon fragments on the *D. melanogaster* and *D. erecta* F elements (46% and 45%, respectively). The high level of DINE-1 in *D. mojavensis* suggests a recent expansion.

To ensure that the low transposon density found on the *D. grimshawi* F element is not an artifact of misassemblies in the CAF1 genome assembly (see File S1), we performed an additional repeat analysis using the species-specific ReAS libraries previously produced by the *Drosophila* 12 Genomes Consortium (*Drosophila* 12 Genomes Consortium *et al.* 2007). ReAS is less susceptible to the effects of misassemblies compared with alignment-based *de novo* repeat finders because it identifies repeats by finding overrepresented sequences within genomic reads (Li *et al.* 2005). This analysis did not alter the conclusion that the *D. grimshawi* F element has the lowest transposon density among the species analyzed here (Figure S4).

#### Multiple subfamilies of the DINE-1 element are observed:

The RepeatMasker results show that most of the differences in the transposon density of the F elements can be attributed to the DINE-1 element (Figure 3D). Comparison of the DINE-1 fragments identified by RepeatMasker using the species-specific libraries *vs.* the RepBase *Drosophila* library (Jurka *et al.* 2005) shows that there are additional DINE-1 elements in the *D. grimshawi*, *D. mojavensis*, and *D. erecta* species-specific transposon libraries that are not in the *Drosophila* RepBase library. Analysis of the distribution of the DINE-1 elements shows that 40% of the DINE-1 fragments (based on total size) on the *D. grimshawi* F and D elements, and 29% on the *D. mojavensis* D element found by the species-specific repeat libraries do not overlap with repeats in the *Drosophila* RepBase library. In contrast, although the *D. mojavensis* F element appears to have an expanded number of DINE-1 elements, only 9% do not overlap with repeats in the *Drosophila* RepBase library (Table S5 and File S5). Analysis of the scaffolds assembled from unmapped *D. mojavensis* modENCODE RNA-Seq reads suggests that some of these helitrons are being transcribed in the *D. mojavensis* genome; a potential candidate is shown in Figure S5. (See File S1 for a more detailed description of this analysis.)

#### Overall repeat distribution on the F element:

Collectively, the repeat analysis shows the F elements have a higher repeat density than the euchromatic reference regions in all four *Drosophila* species. It also shows that although the *D. mojavensis* and *D. grimshawi* F elements have similar total repeat densities, they have strikingly different repeat compositions. A total of 75% of the repeats that overlap with a repeat identified by WindowMasker on the *D. mojavensis* F element are transposons (particularly DINE-1 elements) compared to only 27% on the *D. grimshawi* F element, whereas the *D. grimshawi* F element shows a greater density of simple and low complexity repeats than the *D. mojavensis* F element (39% *vs.* 20%). These differences in repeat composition could impact the local chromatin structure and thus the evolution of the resident genes.

### Evolution of F element genes

Despite its high repeat density, the distal arm of the *D. melanogaster* F element contains 79 genes, many of which have important developmental and housekeeping functions (Riddle *et al.* 2012). Our manual gene annotations (described previously) show that the *D. melanogaster*, *D. erecta*, *D. mojavensis*, and *D. grimshawi* F elements all have approximately 80 genes. The gene density of the F element is lower than that of the euchromatic reference regions from the D element (~60 genes/Mb *vs.* ~80 genes/Mb) for these four species (Table S6). Among the four species, the *D. mojavensis* F element has the lowest gene density (48 genes/Mb compared with 60–66 genes/Mb in the other F elements). This reflects the increased size of the *D. mojavensis* F element due to the expansion of repetitious elements (1.7 Mb *vs.* 1.2–1.3 Mb in the other F elements) (Table S6 and Figure 3).

Although we have produced annotations for all isoforms, our analysis below is based only on the isoform with the largest coding region (*i.e.*, the most comprehensive isoform) for each gene. Restricting our analysis to the most comprehensive isoform allows us to avoid counting the same region multiple times because of alternative splicing. We initially examined genes at the base, extended, and telomeric regions (described previously) of the *D. erecta* D element. Since the genes in these three euchromatic regions exhibit similar characteristics, the primary focus of the following analysis is on the comparison of genes between the F element and the base of the D element (results for all of the analysis regions are available in Figure S6). Summary statistics for all of the gene characteristics, and results of multiple comparison tests after the Kruskal-Wallis (KW) rank sum tests (Kruskal and Wallis 1952), are available in File S6.

#### F element genes are larger because they have larger introns and more coding exons:

**Figure 5:**
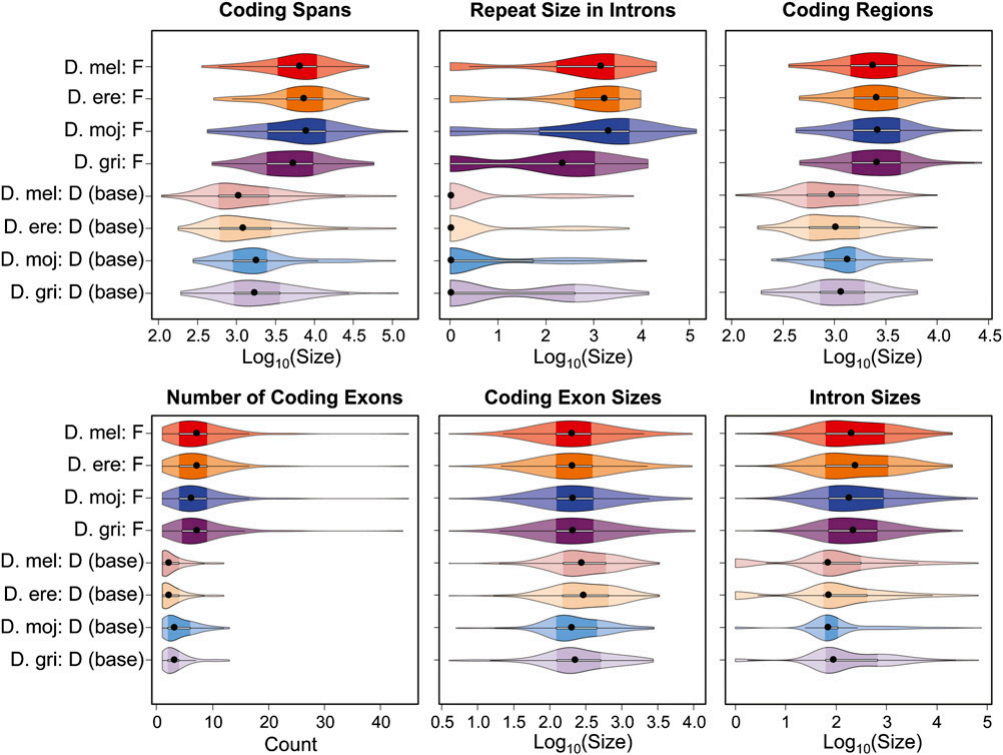
Violin plots of gene characteristics for each analysis region. A violin plot is composed of a boxplot and a kernel density plot: the black dot denotes the median; the darker regions and the thin white box denote the range between the first (Q1) and third (Q3) quartiles [*i.e.*, the interquartile range (IQR)]. Whiskers extending from the white box span from Q1-1.5☓IQR to Q3+1.5☓IQR; the data points beyond the whiskers are outliers. For violin plots using a log scale, a pseudocount of one was added to all data points. The larger coding spans of F element genes can be attributed not only to larger introns (often containing repeats), but also to larger coding regions. The larger coding regions reflect the higher number of coding exons.

Comparisons of the distribution of gene characteristics using violin plots (Hintze and Nelson 1998) show that the coding span (*i.e.*, the region that spans from the start codon to the stop codon, including introns) for F element genes is much larger (median 5156–7569 bp) than for genes at the base of the D elements (median 1028–1736 bp) (Figure 5, top left). The KW test shows that this difference is statistically significant (p-value: 2.12E-48).

Part of this difference in the coding span can be attributed to the significantly higher transposon density (KW test p-value: 2.40E-82) within the introns of F element genes (Figure 5, top center; “repeat size” is the total size of the transposon fragments within the introns of a gene, in bp). Among the four species analyzed in this study, 71–83% of the F element genes contain at least one transposon fragment in an intron. In contrast, only 20–46% of the D element genes contain at least one transposon fragment. Consistent with the results of the transposon density analysis, we find that the *D. mojavensis* F element has the highest intron transposon density (median 1930 bp) whereas *D. grimshawi* has the lowest (median 210 bp).

In addition to differences in the repeat sizes within introns, the violin plots also show that the coding regions (*i.e.*, the region that spans from the start codon to the stop codon, excluding introns) of F element genes are significantly larger (median 2313–2565 bp) than the coding regions for D element genes (median 918–1305 bp) (Figure 5, top right). The KW test shows that this difference in the size of the coding regions is statistically significant (p-value = 7.03E-33). Furthermore, although the actual genes found at the base of the D elements of *D. mojavensis* and *D. grimshawi* differ from those found at the base of the *D. melanogaster* and *D. erecta* D elements (due to various rearrangements), a multiple comparison test after KW shows no significant difference in the size of the coding regions.

To further analyze the difference in the distribution of coding spans and the coding regions between the genes on the F and D elements, we examined the distributions of the number of exons, the coding exon sizes, and intron sizes. Previous analysis has shown that *D. melanogaster* F element genes have more transcribed exons than genes in other domains (Riddle *et al.* 2012). In congruence with this observation in *D. melanogaster*, our analysis shows that F element genes in the four *Drosophila* species have significantly more coding exons (median 6–7) than D element genes (median 2–3) (KW test p-value = 5.59E-50) (Figure 5, bottom left). In contrast, the distributions of coding exon sizes are similar between F element genes (median 196–201.5 bp) and D element genes (median 195–284.5 bp). A KW test indicates that there is a significant difference in the distribution of coding exon sizes (p-value = 2.12E-07). However, multiple comparison tests show that only the differences between the coding exons of all four F elements and the coding exons from the base of the *D. melanogaster* and *D. erecta* D elements are statistically significant (see File S6). Hence, in general, F element genes have larger coding regions because they tend to have more coding exons than D element genes.

Consistent with the greater transposon density on the F element, we find that F element genes generally have significantly larger introns (median 172.5–228 bp) than D element genes (median 65–84 bp) (Figure 5, bottom right; KW test p-value = 6.14E-62). Multiple comparison tests show that *D. grimshawi* is the exception, as the difference in intron sizes between the *D. grimshawi* F and D element genes is not statistically significant. The intron size distribution for the *D. grimshawi* D element is significantly different from that of the other D elements, but is not significantly different from that of the *D. melanogaster* and *D. erecta* F elements. These observations are in concordance with the results of the transposon density analysis, which shows that the *D. grimshawi* F and D elements have more similar transposon densities compared to those of other species (see Figure 3D).

Hence the larger coding spans observed for F element genes (Figure 5, top left) can primarily be attributed to a combination of significantly larger repeat sizes within introns (Figure 5, top center) and larger coding regions (Figure 5, top right). The larger coding regions of F element genes can be attributed to a significantly higher number of coding exons (Figure 5, bottom left) but not to the size of the individual coding exons (Figure 5, bottom center). Introns of F element genes are significantly larger than introns of genes in the euchromatic reference regions for *D. melanogaster*, *D. erecta*, and *D. mojavensis* but not for *D. grimshawi* (Figure 5, bottom right).

#### F element genes show lower codon bias than D element genes:

Previous analysis of codon usage bias in 12 *Drosophila* species (using 33 *D. melanogaster* F element genes and their corresponding GLEAN-R annotations) showed that F element genes exhibit lower codon bias compared with genes on the other Muller elements (Vicario *et al.* 2007). Here we expand the codon bias analysis to all of the manually annotated F element genes in four *Drosophila* species using two metrics: the Effective Number of Codons (Nc), which measures deviations from uniform codon usage (Wright 1990), and the CAI, which measures deviations from the species-specific optimal codon usage (Sharp and Li 1987). (Lower Nc values and higher CAI values indicate stronger codon bias.)

**Figure 6:**
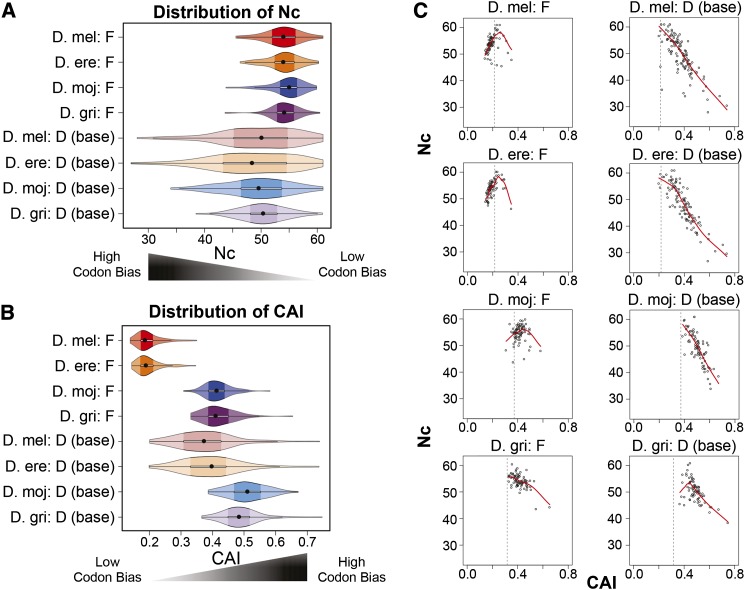
F element genes exhibit different patterns of codon bias in *D. mojavensis* and *D. grimshawi* compared to *D. melanogaster* and *D. erecta*. (A) Distributions of Effective Number of Codons (Nc). (B) Distributions of Codon Adaptation Index (CAI). (C) Scatterplots of Nc *vs.* CAI show that, similar to the base of the D elements, codon bias in the *D. grimshawi* F element genes can be attributed primarily to selection rather than mutational biases, as indicated by a LOESS regression line (red line) with negative slope (see main text). The dotted line in each Nc *vs.* CAI scatterplot demarcates the CAI value for a gene with no codon bias relative to the species-specific reference gene sets constructed by the program scnRCA (see File S1).

Violin plots of Nc show that F element genes exhibit significantly smaller deviations from uniform codon usage (median 53.92–54.95) than genes at the base of the D elements (median 48.35–50.33) in all four species (KW test p-value = 8.84E-38) (Figure 6A). Multiple comparison tests show that the contrast between F and D genes is the only statistically significant difference in the distribution of Nc. Violin plots of CAI also show that F element genes exhibit significantly lower codon bias than D element genes (KW test p-value = 1.66E-119) (Figure 6B). However, multiple comparison tests show that the CAIs for *D. mojavensis* and *D. grimshawi* are significantly greater (indicating more optimal codon usage) than those for *D. melanogaster* and *D. erecta* for both the F element genes (median 0.409–0.412 *vs.* 0.185–0.188) and the D element genes (median 0.483–0.510 *vs.* 0.372–0.397).

#### Codon bias in D. grimshawi F element genes can primarily be attributed to selection:

To infer the selective pressure experienced by genes in the different analysis regions, we compared the Nc and CAI values of each gene using a scatterplot (Vicario *et al.* 2007). This analysis posits that Nc measures deviations from uniform codon usage that could either be attributed to mutational bias or selection, while CAI measures deviations from optimal codon usage and primarily reflects selection. Hence, genes that exhibit both large deviations from uniform codon usage (*i.e.*, low Nc) and small deviations from optimal codon usage (*i.e.*, high CAI) are thought to be under stronger selective pressure, while genes with low Nc and low CAI are under stronger influence from mutational biases (Vicario *et al.* 2007). After constructing the Nc *vs.* CAI scatterplots for each analysis region, we applied locally estimated scatterplot smoothing (LOESS, (Cleveland and Devlin 1988)) to capture the overall trends seen in each scatterplot (Figure 6C). Regression lines that show a positive slope indicate that the codon bias can primarily be attributed to mutational biases, while a negative slope indicates that the codon bias can primarily be attributed to selection on codon usage.

Consistent with previous reports using a smaller gene set (Vicario *et al.* 2007), our analysis shows that codon bias for most of the genes on the *D. melanogaster* and *D. erecta* F elements can be attributed to mutational biases rather than selection (*i.e.*, most of the genes are in the part of the LOESS regression line that shows a positive slope), indicating low selective pressure relative to what is seen for the D element genes. In contrast, we find that codon bias for most of the genes on the *D. grimshawi* F element, along with genes on the D elements, can primarily be attributed to selection (*i.e.*, most of the genes are in the part of the LOESS regression line with negative slope). Thus we observe that the F element with the lowest transposon density (*D. grimshawi*) differs from the other F elements in this regard, with more of the genes showing evidence of response to selective pressure. We also find that most of the *D. mojavensis* F element genes have CAI values that are higher than those for a gene with equal codon usage (dotted line in Figure 6C), indicating a more optimal pattern of codon usage compared to F element genes in *D. melanogaster* and *D. erecta*. Although most of the F element genes within each Nc *vs.* CAI scatterplot follow a similar trend, there are a few outliers (Figure 6C). For example, the Muller F element genes *ATPsyn-beta* and *RpS3A* exhibit low Nc and high CAI in all four *Drosophila* species (Figure S7, see *Discussion*) (See File S1 and the heat maps in File S7 for the detailed analysis on the changes in codon usage preferences for each amino acid).

#### A subset of F element genes exhibits distinct characteristics in all four species:

Our analyses show that the overall characteristics of F element genes are distinct from genes at the base of the D element. However, previous studies have shown that some regions on the *D. melanogaster* F element differ from the general case in being enriched in H3K27me3, rather than H3K9me2/3, in a tissue-specific fashion; genes that reside in these regions are associated with *Polycomb* (PcG) (Kharchenko *et al.* 2011; Riddle *et al.* 2012). PcG proteins regulate the expression of many genes involved in development (such as homeotic genes) by altering the chromatin structure (reviewed in (Lanzuolo and Orlando 2012)). Hence it is of particular interest to ask whether the six F element genes associated with PcG exhibit characteristics that differ from the rest of the F element genes.

**Figure 7:**
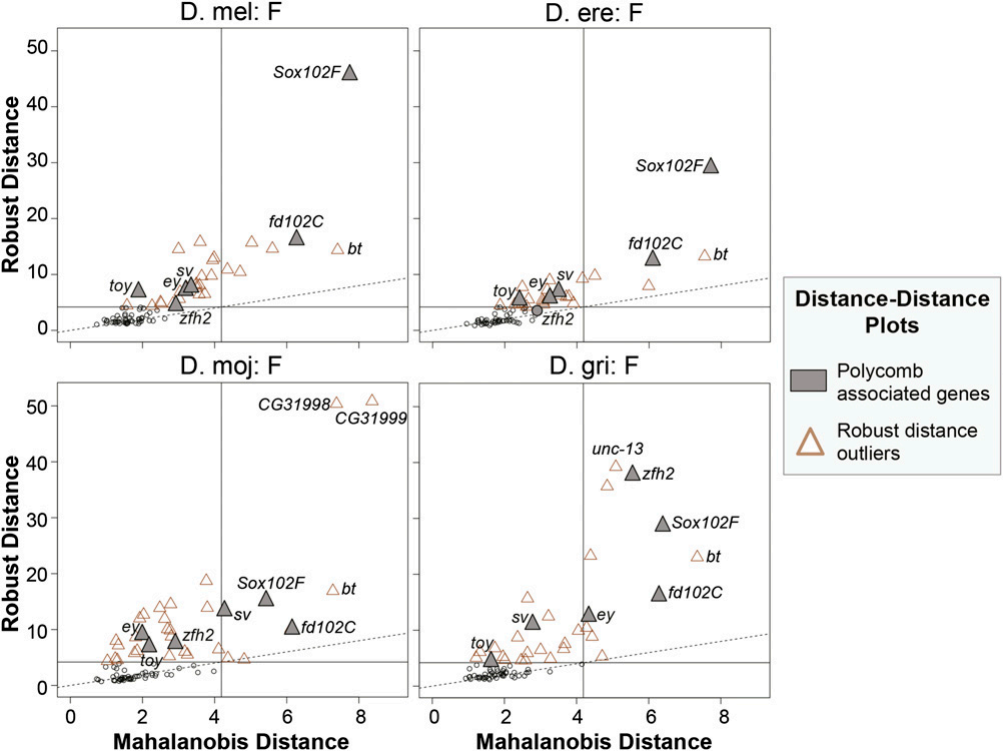
Distance–Distance Plots of robust distance (RD) *vs.* Mahalanobis distance (MD) show both common and species-specific outliers. The horizontal and vertical lines correspond to the cutoff values for outliers (97.5% quantile of the χ^2^ distribution, see File S1). Values greater than the cutoff values identify outliers. Triangles in the upper right quadrant are outliers based on both RD and MD. Triangles in the upper left quadrant are outliers only based on RD. The dashed line corresponds to points with equal RD and MD values. F element genes that reside in a *Polycomb* domain in *D. melanogaster* are highlighted in gray.

Because there are only six genes on the *D. melanogaster* F element that are associated with PcG, there is insufficient statistical power to analyze each gene characteristic separately to ascertain if PcG genes exhibit significantly different properties compared to the other F element genes. Consequently, we performed a multivariate analysis of the gene characteristics described above (see File S1 for details). For each F element, we constructed a Distance–Distance (DD) plot (Rousseeuw and Van Zomeren 1991) of gene characteristics to identify outliers (Figure 7). Detection of outliers using MDs (Mahalanobis 1936) show that there are three F element genes (*bt*, *fd102C*, and *Sox102F*) that consistently exhibit characteristics that are distinct from other F element genes in all four species. The *bt* gene, for example, is an outlier because it has a substantially larger coding span, larger coding region, and more coding exons compared to the other F element genes in all four species. The DD plot also identifies some species-specific outliers: *CG31999* is an outlier in the *D. mojavensis* F element because it has a gene size of 157 kb (compared to 10 kb in *D. melanogaster*).

Detection of outliers using robust distances identifies additional outliers (triangles in the top left quadrant, Figure 7) that are not detected by the MD because of the masking effect (Ben-Gal 2005). Robust distance in the DD plots identifies 25–29 F element genes as outliers and 14 of these outliers are found in all four species. Analysis of these 14 genes using modMINE (Contrino *et al.* 2012) shows that they are significantly enriched in “RNA polymerase II distal enhancer sequence specific DNA binding transcription factor activity” (GO:0003705, Holm-Bonferroni adjusted p-value = 8.36E-4).

Of the 14 outliers that are found in all four species, five of them (*ey*, *fd102C*, *Sox102F*, *sv*, and *toy*) are associated with PcG domains. The only exception is *zfh2*, which is an outlier in three of the four species (*D. melanogaster*, *D. mojavensis*, and *D. grimshawi*). Hence the DD plot analysis suggests that F element genes that reside in domains enriched in H3K27me3 might have different characteristics than F element genes that reside in domains enriched in H3K9me2/3.

### F element genes show lower melting temperature metagene profiles

Despite residing in a domain with heterochromatic properties, *D. melanogaster* F element genes exhibit expression levels that are similar to those of other euchromatic genes (Riddle *et al.* 2012). One of the mechanisms for regulating gene expression is the pausing of RNA Polymerase II during early elongation (reviewed in (Adelman and Lis 2012)). Previous analysis has shown that the efficacy of elongation depends on the stability of the 9-bp RNA-DNA hybrid in the elongation complex (Tadigotla *et al.* 2006). Genes that exhibit polymerase pausing have a distinct 9 bp melting temperature profile (*i.e.*, greatest melting temperature at 25–30 bp downstream of the transcription start site, where pausing occurs) (Nechaev *et al.* 2010).

Previous studies have shown that *D. melanogaster* F element genes exhibit lower melting temperatures than genes that reside in other domains (Riddle *et al.* 2012). To ascertain whether this difference is conserved in other *Drosophila* species, we performed a metagene analysis of the melting temperature profile. (See the section *Materials and Methods* for details on the definition of the metagene.)

**Figure 8:**
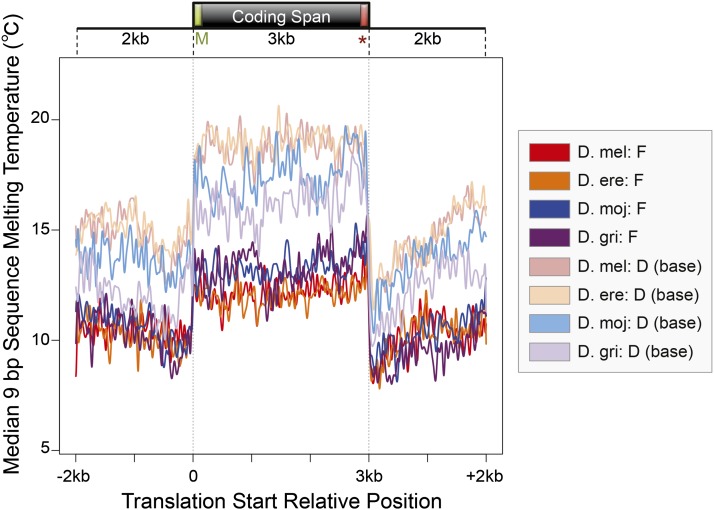
Metagene analyses show that F element genes have a lower median 9-bp melting temperature (*T_m_*) than genes at the base of the D element. The 9-bp *T_m_* was calculated using a sliding-window of 9 bp and a step size of 1 bp. The *T_m_* for each coding span was subsequently normalized to 3 kb to create the metagene profile (see File S1).

The metagene profiles show that F element genes in all four *Drosophila* species have lower melting temperatures (T_m_) than genes at the base of the D element. In all cases, the coding spans (*i.e.*, from start codon to stop codon, including introns) show substantially higher melting temperatures than the 2-kb flanking regions (Figure 8). Coding spans of the *D. mojavensis* and *D. grimshawi* F elements show greater *T_m_* than those of *D. melanogaster* and *D. erecta*. Comparing the F element and D element genes within a given species, we find that those of *D. grimshawi* show the smallest difference in the melting temperature profiles.

### F element gene rearrangements and gene movements

#### Changes in F element gene order:

Previous studies have estimated that approximately 95% of the genes in *D. melanogaster* remain on the same Muller element across the 12 *Drosophila* species (Bhutkar *et al.* 2008). To ascertain whether the low rate of recombination would affect the rate of rearrangements and gene movements on the F element, we analyzed the placement of *D. melanogaster* F element genes in the other *Drosophila* species.

Of the 79 *D. melanogaster* F element genes annotated by FlyBase, two of the genes were omitted from the gene movement analysis because they are either a partial gene (*JYalpha*) or a possible misannotation (*CG11231*). (See File S1 for details.) Of the remaining 77 *D. melanogaster* F element genes, all 77 genes (100.0%) are found on the *D. erecta* F element, 72 (93.5%) are found on the *D. mojavensis* F element and 73 (94.8%) are found on the *D. grimshawi* F element.

Except for *CG11231*, the *D. erecta* F element is completely syntenic with respect to the *D. melanogaster* F element. GRIMM (Tesler 2002) estimates that a minimum of 31 inversions are required to transform the *D. melanogaster* F element gene order and orientation to that observed in the *D. mojavensis* F element (72 genes in common). Similarly, at least 33 inversions are required to transform the *D. melanogaster* F element gene order to that observed in *D. grimshawi* (73 genes in common). There are 78 genes that are found on both the *D. mojavensis* and *D. grimshawi* F elements, and GRIMM estimates a minimum of seven inversions are required to transform the gene order in *D. mojavensis* to that observed in *D. grimshawi*. (See possible rearrangement scenarios estimated by GRIMM in Figure S8.)

Analysis of the number of genes per syntenic block (*i.e.*, syntenic block sizes) shows that the F elements have smaller syntenic blocks than the previously reported genome averages (Bhutkar *et al.* 2008). The *D. mojavensis* F element has an average syntenic block size of 3.4 genes compared to an average of 8.8 genes per syntenic block for the whole genome. The corresponding numbers for *D. grimshawi* are 3.6 and 8.4 genes per syntenic block for the F and D elements, respectively. Thus inversions are common on the F element despite its low rate of recombination.

#### Identifying a wanderer gene hotspot:

**Figure 9:**
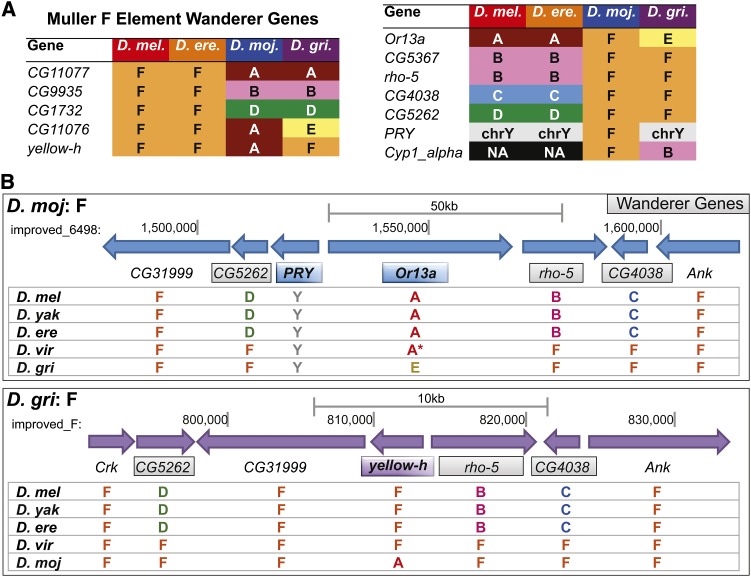
F element gene movements in the four *Drosophila* species analyzed in this study. (A) Placement of the 12 F element wanderer genes [five on the F element in *D. melanogaster* and *D. erecta* (top left), and seven on the F element in *D. mojavensis* (top right)]. (B) Schematic representations of the wanderer gene hotspots on the *D. mojavensis* and *D. grimshawi* F elements where most of the wanderer genes are found. The genes *PRY* and *Or13a* (blue boxes) have moved from other Muller elements to the *D. mojavensis* F element. The gene *yellow-h* (purple box) has moved from the F element to the A element in *D. mojavensis*. Assignment of the *D. virilis* ortholog of *Or13a* to the A element (denoted by an asterisk) is based on the placement of the other seven genes found in that scaffold (13050) (see File S1). Placement of *PRY* on the Y chromosome is based on Koerich *et al.* (2008).

Movement of genes between different chromosomes typically results from gene duplications (via ectopic recombination or retrotransposition) followed by the loss of the original copy of the gene (Meisel *et al.* 2009). There are 12 genes that are found on the F element in one *Drosophila* species, but on another Muller element in a different *Drosophila species* (“wanderer genes”; Figure 9A). One of these wanderer genes is a putative paralog of *Cyp1* (*Cyp1_alpha*) that is found on the *D. mojavensis* F element and the *D. grimshawi* B element but is not found in either *D. melanogaster* or *D. erecta*.

To further analyze the distribution of wanderer genes on the F elements, we compared the genome assemblies of six *Drosophila* species (*D. melanogaster*, *D. yakuba*, *D. erecta*, *D. virilis*, *D. mojavensis*, and *D. grimshawi*) using the UCSC Chain and Net protocol (Kent *et al.* 2003). Examination of the Net alignment tracks shows there is a single region (*i.e.*, hotspot) in both the *D. mojavensis* and *D. grimshawi* F elements where most of the wanderer genes are found (Figure S9). The *D. mojavensis* F element hotspot contains five of the six wanderer genes relative to *D. melanogaster* (Figure 9B, top). The hotspot on the *D. grimshawi* F element contains three of the four wanderer genes relative to *D. melanogaster* and one of the wanderer genes (*yellow-h*) relative to *D. mojavensis* (Figure 9B, bottom).

Because three of the wanderer genes (*CG5262*, *rho-5*, and *CG4038*) are found in the wanderer gene hotspots of both the *D. mojavensis* and *D. grimshawi* F elements (relative to *D. melanogaster*), we can use them to infer the direction of gene movements of the rest of the wanderer genes in the hotspot. The *yellow-h* gene likely moved from the F element to the A element in *D. mojavensis*. In contrast, both the *PRY* and *Or13a* genes likely moved from other chromosomes (the Y chromosome and the A element, respectively) to the *D. mojavensis* F element. Hence our analysis indicates that gene movement occurs in both directions on the F element and that the cumulative effect of these gene movements is that there are a similar number of genes (~80) on the F element in all four species.

## Discussion

### F elements exhibit distinct characteristics in *Drosophila*

The *D. melanogaster* F element is unusual in that it appears to be predominantly heterochromatic, but in the distal 1.3 Mb has a gene density similar to the euchromatic chromosome arms (Sun *et al.* 2004; Riddle *et al.* 2011, 2012). Immunofluorescent staining of the polytene chromosomes shows that the *D. melanogaster*, *D. erecta*, *D. mojavensis*, and *D. grimshawi* F elements are enriched in H3K9me2 (Figure 1), which suggests that the F element is generally packaged as heterochromatin in these four species. In order to elucidate the impact of these unusual characteristics on the evolution of the F element and its genes, we performed a comparative analysis of the F elements and euchromatic regions near the base of the D elements (coordinates listed in Table S1).

To increase the accuracy of our analysis, we improved the assemblies of the *D. mojavensis* and *D. grimshawi* F elements and the base of the *D. mojavensis* D element, closing 72 of 86 gaps and adding 44,468 bases to these assemblies (Figure 2 and Table S2). Restriction digests and consistent mate pairs provide strong experimental support for the final assemblies. We also produced gene annotations for the regions under study in *D. erecta*, *D. mojavensis*, and *D. grimshawi* (878 genes, 1619 isoforms). Each gene was annotated at least twice independently and reconciled by a third investigator, giving increased confidence in the results. We find substantial differences between our manually curated gene models and the GLEAN-R gene predictions, with only 32–58% of the GLEAN-R gene models showing complete congruence in the cases of *D. mojavensis* and *D. grimshawi* (Table S3). These results illustrate the benefits of manual sequence improvement and gene annotations for regions with moderate repeat density.

Our analysis shows that the F element has generally maintained its distinct characteristics compared with the other autosomes in species that diverged from *D. melanogaster* 40–60 million years ago. Compared with the euchromatic reference regions within each species, we find that F elements have higher repeat density (Figure 3 and Figure 4), and the genes are larger, have larger introns, more coding exons (Figure 5), lower codon bias (Figure 6), and lower melting temperatures (Figure 8). Most F element genes exhibit similar characteristics within each species but there are also species-specific and common outliers among the four *Drosophila* species (Figure 7). Analysis of gene movements shows that the F elements have smaller syntenic blocks than the genome average and that there is a single hotspot in both the *D. mojavensis* and *D. grimshawi* F elements where most of the wanderer genes are found (Figure 9). We also identified genes that have moved both on and off of the F element, maintaining approximately the same number of genes in the four species. It is striking that these gene movements (presumably due to transposition) occur at a rate similar to that seen for the other autosomes, and inversions are more frequent, while recombination is reduced. This suggests that the frequency of such events is not dictated solely by DNA accessibility, as such a simple model of the consequences of heterochromatin packaging might have been thought to impact all three types of events equally.

Although the F elements generally show similar characteristics, we also find some differences among the four *Drosophila* species (particularly between the species in the *Drosophila* clade *vs.* the species in the melanogaster subgroup of the *Sophophora* clade). These differences could provide insights into the impact of low recombination rate on the evolution of the genomic landscape (*e.g.*, repeats and gene characteristics) of the F element.

### F elements have different repeat compositions

One of the prominent characteristics of heterochromatin is its high repeat density. Previous studies have shown that the difference in total repeat density is one of the major contributors to the changes in genome size among the different *Drosophila* species (Bosco *et al.* 2007). A critical consideration here is that some classes of transposons and tandem repeats have been implicated in gene silencing and heterochromatin formation (Martienssen 2003; Riddle *et al.* 2008; Sentmanat and Elgin 2012).

In concordance with previous reports for many eukaryotes (Tóth *et al.* 2000), our dinucleotide repeat analysis shows a lack of CG dinucleotide repeats on both the F and D elements in all four *Drosophila* species (Figure 4B). Previous studies have shown that there is a strong mutational bias in *Drosophila* toward A/T, whereas codon bias tends to favor G/C at synonymous sites (Moriyama and Powell 1997; Vicario *et al.* 2007). Hence the lack of CG dinucleotide repeats on the F element could be explained by its low recombination rate. However, this mutational bias does not explain the lack of CG dinucleotide repeats on the D elements. Previous studies have shown that methylated CpG sequences have a greater rate of mutation because they are susceptible to spontaneous deamination, and the low frequency of CG repeats has been attributed to this (Duncan and Miller 1980). Hence, the lack of CG dinucleotide repeats on the D element is striking given the low levels (if any) of DNA methylation in *Drosophila* (Raddatz *et al.* 2013; Takayama *et al.* 2014). Another explanation for the lack of CG repeats is clearly needed.

Previous *in situ* hybridization analyses by Pardue *et al.* (1987) have shown that CA/GT dinucleotide repeats are highly enriched on the *D. melanogaster* X chromosome but are depleted in the F element and β-heterochromatin (*i.e.*, heterochromatin that is replicated during polytenization). In contrast, the *D. virilis* F element is enriched in CA/GT dinucleotide repeats (Pardue *et al.* 1987). Our analysis shows that, similar to *D. virilis*, the *D. mojavensis* and *D. grimshawi* F elements have long CA and AG dinucleotide repeats, whereas the *D. melanogaster* F element is notably depleted in these dinucleotide repeats (Figure 4B). However, the significance of these differences in the distribution of dinucleotide repeats is unclear.

Our analysis also shows that the *D. mojavensis* and *D. grimshawi* F elements contain longer AT dinucleotide repeats than *D. melanogaster* and *D. erecta* (Figure 4B). Previous analyses have shown that long AT dinucleotide repeats inhibit the formation of nucleosomes (reviewed in (Struhl and Segal 2013)). Hence, this difference in the frequency of long AT dinucleotide repeats suggests that the *D. mojavensis* and *D. grimshawi* F elements might not be as densely packaged as the *D. melanogaster* and the *D. erecta* F elements.

Estimates of the total repeat content with WindowMasker show that the *D. mojavensis* and *D. grimshawi* F elements have similar repeat density and both species have a higher repeat density than the *D. melanogaster* and *D. erecta* F elements (Figure 3A). However, the *D. mojavensis* and *D. grimshawi* F elements have different repeat compositions: most of the repeats in the *D. mojavensis* F element (~75%) are transposons whereas more of the repeats (~39%) in the *D. grimshawi* F element are simple and low complexity repeats.

Among the four species, *D. mojavensis* has the greatest F element transposon density (50%), whereas *D. grimshawi* has the lowest (20%). The differences in transposon density can primarily be attributed to changes in the density of the DINE-1 element (27% in *D. mojavensis vs.* 2% in *D. grimshawi*) (Figure 3D). The DINE-1 element was first characterized in *D. melanogaster* and this transposon is primarily found on the F element and in pericentric heterochromatin (Locke *et al.* 1999). Subsequent studies have classified the DINE-1 as a helitron, and have shown that there has been a more recent transposition and expansion of DINE-1 elements in *D. yakuba* and *D. mojavensis*, which results in the higher density of DINE-1 elements in these species. In contrast, the *D. grimshawi* genome has the lowest density of DINE-1 elements among the 12 *Drosophila* species, possibly because it is geographically isolated (on the Hawaiian islands) and might not have experienced the same transpositional burst of the DINE-1 elements seen in many of the other *Drosophila* species (Yang *et al.* 2006; Yang and Barbash 2008).

In concordance with previous reports (Kuhn and Heslop-Harrison 2011), comparison of the overlap between the DINE-1 fragments identified by the species-specific transposon library and the *Drosophila* RepBase library indicates that there are at least two major subfamilies of DINE-1 elements in *D. mojavensis* (Table S5). We found that 67% of the DINE-1 fragments in the species-specific library overlap with the Homo6 transposon whereas 22% overlap with the Helitron1_Dmoj transposon (File S5). Analysis of the *D. mojavensis* RNA-Seq data (Graveley *et al.* 2011) identified a scaffold that contains a conserved Helitron_like_N (Pfam accession: PF14214) domain (Figure S5), indicating that some of the DINE-1 elements may still be active. A transposable element present at a high density, in a genome that expresses that transposable element, could well be a target for silencing, promoting heterochromatin formation.

The horizontal transfer and subsequent amplification of helitrons occur in many organisms, including mammals, reptiles, and insects (Thomas *et al.* 2010). Helitrons can capture adjacent gene fragments during transposition and can affect the evolution of the host species [reviewed in (Kapitonov and Jurka 2007)]. Previous analysis of 12 *Drosophila* species shows that DINE-1 fragments often are found in introns or within 1 kb of the coding regions (Yang and Barbash 2008). Hence the DINE-1 element may play an important role in shaping the genomic landscape of the F elements and their genes.

The high repeat density of the F element has a direct impact on gene characteristics. One of the factors that contributes to the significantly larger coding span of F element genes compared to D element genes is that F element genes have significantly larger introns in all of the species examined here except for *D. grimshawi* (Figure 5, lower right). This difference in intron size can partly be attributed to the differences in intron repeat density (Figure 5, top center). However, this does not *a priori* explain the other factor contributing to the larger coding span of F element genes — the larger number of coding exons.

### The *D. grimshawi* F element genes exhibit different patterns of codon bias

A salient characteristic of the F element is its low rate of recombination (Arguello *et al.* 2010; Wang *et al.* 2002). Codon bias is correlated with the recombination rate because of the Hill-Robertson effect (Hill and Robertson 1966; Kliman and Hey 1993). In agreement with this effect, we find that F element genes exhibit lower codon bias than D element genes based on both the Nc and the CAI metric (Figure 6).

Although F element genes for all four species exhibit smaller deviations from uniform codon usage (*i.e.*, low Nc) than D element genes, we find that *D. mojavensis* and *D. grimshawi* genes show a more optimal pattern of codon usage (*i.e.*, greater CAI) than *D. melanogaster* and *D. erecta* genes in both the F and D elements. The greater CAIs in the *D. mojavensis* and *D. grimshawi* analysis regions are in congruence with the results from previous whole genome analysis of CAIs in 12 *Drosophila* species, which shows that the distribution of CAIs for species in the *Drosophila* subgroup are shifted to the right (*i.e.*, greater CAI) compared with the melanogaster subgroup (Heger and Ponting 2007).

In concordance with the hypothesis that greater CAI reflects stronger selection because of greater transfer RNA abundance (Moriyama and Powell 1997) and greater expression levels (Duret and Mouchiroud 1999), we find that the F element genes *ATPsyn-beta* and *RpS3A* exhibit strong codon bias in all four *Drosophila* species (Figure S7). *ATPsyn-beta* is an ATPase (Peña and Garesse 1993) and *RpS3A* is a ribosomal protein (Van Beest *et al.* 1998). Both genes are very highly expressed in all developmental stages in *D. melanogaster* (Graveley *et al.* 2011).

Scatterplots of Nc *vs.* CAI can indicate whether the codon bias observed in each region can primarily be attributed to mutational bias or selection (Vicario *et al.* 2007). Unlike those of the other F elements, the *D. grimshawi* F element genes show a negative correlation between Nc and CAI, similar to the D element genes (Figure 6). Thus, in contrast to the other F elements, more of the codon bias in *D. grimshawi* F element genes can be attributed to selection rather than mutational biases.

The results of the repeat density and codon bias analyses suggest that the *D. grimshawi* F element has a greater rate of recombination. This might be a consequence of the lower transposon density, given that transposons can be targets for heterochromatin formation (Lippman and Martienssen 2004; Sentmanat *et al.* 2013). Furthermore, the low density of DINE-1 elements on the *D. grimshawi* F element compared with the other species suggests that this transposon might play an important role in promoting heterochromatin assembly. However, the transposon families present vary in the different species, and there may well be other transposable elements, present in other species but absent from *D. grimshawi* [*e.g.*, *1360* and *Galileo*, (Marzo *et al.* 2008)] that could contribute substantially to silencing.

### Lower melting temperatures may facilitate transcription of F element genes

Previous analysis has shown that a much smaller fraction of the *D. melanogaster* F element genes exhibit polymerase pausing (1.6%) compared with genes found in pericentric heterochromatin (12.5%) or euchromatin (15.0%). F element genes also show a lower melting temperature near the transcription start site than genes in the other *D. melanogaster* Muller elements, irrespective of whether the genes exhibit polymerase pausing (Riddle *et al.* 2012). Our metagene analysis of melting temperature profiles shows that F element genes in all four *Drosophila* species exhibit lower melting temperatures across the entire span of the metagene than D element genes (Figure 8). The lower melting temperature suggests that, similar to *D. melanogaster*, only a small fraction of the *D. erecta*, *D. mojavensis*, and *D. grimshawi* F element genes will exhibit polymerase pausing.

The elongation rate of RNA Polymerase II can affect the total mRNA level (Danko *et al.* 2013) and previous studies have found that the rate of elongation is negatively correlated with GC content within the gene body, and with exon density (Jonkers *et al.* 2014). Although F element genes are larger and have more coding exons than euchromatic genes (Figure 5), the metagene has a substantially lower melting temperature (Figure 8), presumably because of the high AT content within introns and the low codon bias. The high AT content in the genes could be a consequence of the less effective selection for codon bias (because of the low rate of recombination on the F elements) coupled with the A/T mutational bias in *Drosophila*. The lower GC content within the gene body could facilitate transcription and hence help explain how F element genes can have expression levels that are similar to genes in euchromatic regions, despite residing in a domain with heterochromatic properties.

This study provides an initial survey of the evolution of the F element and its genes in four *Drosophila* species. Our results show that the F element has maintained its distinct characteristics in both the *Sophophora* and *Drosophila* subgenera. The unusual mixture of a heterochromatic domain with a euchromatin-like gene density on the F element enabled us to investigate a number of interesting questions relating genome organization to gene function. The genomics resources (*e.g.*, improved assemblies, gene annotations, genome browsers) produced in this study provide a foundation for future investigations into the factors that impact chromatin packaging and gene expression in a heterochromatic domain.
